# The conserved *SEN1* DNA/RNA helicase has multiple functions during yeast meiosis

**DOI:** 10.1371/journal.pgen.1011684

**Published:** 2025-12-11

**Authors:** Robert Gaglione, Leonidas Pierrakeas, Lihong Wan, Jonathan Caradonna, Amy J. MacQueen, Ed Luk, Nancy M. Hollingsworth

**Affiliations:** 1 Department of Biochemistry and Cell Biology, Stony Brook University, Stony Brook, New York, United States of America; 2 Department of Molecular Biology and Biochemistry, Wesleyan University, Middletown, Connecticut, United States of America; University College Dublin, IRELAND

## Abstract

DNA:RNA hybrids are unusual structures found throughout the genomes of many species, including yeast and mammals. While DNA:RNA hybrids may promote various cellular functions, persistent hybrids lead to double strand breaks, resulting in genomic instability. DNA:RNA hybrid formation and removal are therefore highly regulated, including by enzymes that either degrade or unwind RNA from the hybrid. Meiosis is the specialized cell division that creates haploid gametes for sexual reproduction. Previous work in yeast and mammals showed that elimination of DNA:RNA hybrids by RNase H facilitates meiotic recombination. This work demonstrates that the conserved Sen1 DNA/RNA helicase functions during three temporally distinct processes during yeast meiosis. First, *SEN1* allows meiosis-specific genes to be expressed at the proper time to allow entry into meiosis. Second, *SEN1* prevents the accumulation of hybrids during premeiotic DNA replication. Third, *SEN1* promotes the repair of programmed meiotic double strand breaks that are necessary to form crossovers between homologous chromosomes to allow their proper segregation at the first meiotic division. Given the evolutionary conservation of Sen1 with its mammalian counterpart, Senataxin, studies of Sen1 function in yeast are likely to be informative about the regulation of DNA:RNA hybrids during human meiosis as well.

## Author summary

Yeast cells undergo meiosis when starved, which triggers expression of Ime1, a master regulatory protein that activates transcription of meiotic “early genes” that mediate several meiotic processes, including recombination and chromosome synapsis. During prophase I, the conserved topoisomerase-like protein, Spo11, creates double strand breaks that are used to identify homologous chromosomes and generate crossovers between them. DNA:RNA hybrids are formed when an RNA molecule anneals to a complementary strand of DNA and are present at the ends of double strand breaks during prophase I of meiosis in a variety of organisms. DNA:RNA hybrids can be removed by degradation of the RNA by RNase H or by unwinding of the RNA by an essential multi-functional DNA/RNA helicase called Sen1, a yeast ortholog of the mammalian Senataxin helicase. Mouse mutants lacking Senataxin exhibit male infertility and defects in double strand break repair. Using a variety of approaches, we show that *SEN1* has multiple temporally distinct roles that promote yeast meiosis. First, it enables the timely expression of *IME1*-regulated early genes. Second, it helps prevent and/or remove DNA:RNA hybrids that form during premeiotic S phase. Third, it facilitates both repair of Spo11-generated double strand breaks generated during prophase I and chromosome synapsis.

## Introduction

DNA:RNA hybrids arise in cells when an RNA molecule base pairs with a complementary single strand of DNA. If the RNA associates with a duplex of DNA, the DNA strand of like polarity is displaced, creating a specific structure called an R-loop. It is estimated that 5–10% of the genome contains DNA:RNA hybrids [1]. DNA:RNA hybrids have beneficial effects in a variety of cellular processes, including transcriptional termination, chromatin structure, kinetochore function at metaphase I of meiosis and repression of antisense transcription [2–5]. However, R-loops that persist can lead to the creation of double strand breaks (DSBs), resulting in genomic instability [6–10]. In addition, DNA:RNA hybrids present at the ends of existing DSBs may either help or hinder DSB repair by promoting resection or blocking recombinases from binding to resected single stranded (ss) ends, respectively [1,11–17]. Furthermore, in some cases, the presence of too few or too many hybrids is deleterious, indicating that the amount of DNA:RNA hybrids must be tightly regulated [3,17].

The importance of regulating DNA:RNA hybrids is also evident by the numerous mechanisms and proteins involved in preventing or removing them [1,18,19]. One way to eliminate DNA:RNA hybrids is degradation of the RNA by the conserved RNase H1 and RNase H2 enzymes [7,20,21]. An alternative mechanism is to unwind the RNA from the DNA using helicases such as the conserved essential 5’-3’ yeast helicase, Sen1, which is orthologous to the mammalian helicase Senataxin [22–25]. The Sen1 protein is multi-functional; in addition to resolving R-loops, Sen1 forms a complex with Nrd1 and Nab3 to mediate transcriptional termination of unstable non-coding RNAs, as well as small nucleolar RNAs. Through this activity, Sen1 influences the distribution of RNA polymerase II across the genome [22].

Given that DNA:RNA hybrids influence the repair of DSBs in vegetatively growing cells, an interesting question is whether they affect DSB repair of the programmed DSBs that initiate recombination during meiosis. Recent work looking at meiosis in mice, nematodes, and yeast defective in RNase H activity supports this idea. Mice in which RNase H1 was specifically knocked down in the germline exhibit male infertility, and spermatocytes display DSB repair defects and prophase I arrest [11]. DNA:RNA hybrids also accumulate in the nematode germline in *rnh-1.0 rnh-2* mutants [26]. Budding yeast strains containing *rnh1Δ rnh201Δ* lack RNase H activity and the resulting defects are exacerbated by deletion of *HPR1*, which disrupts the THO complex that binds to nascent messenger RNAs and assists in their maturation and nuclear export [17,27–29]. Both *rnh1∆ rnh201∆* and *rnh1∆ rnh201∆ hpr1∆* diploids exhibit delayed meiotic progression and reduced spore viability [17,30,31].

Cytological and genomic studies in yeast using the S9.6 antibody to detect DNA:RNA hybrids in mutants lacking RNase H activity showed that hybrids form on ssDNA at the ends of DSBs generated by the meiosis-specific Spo11 protein during prophase I [17,31,32]. Furthermore, in both yeast and mammals lacking RNase H activity, loading of the Rad51 and Dmc1 recombinases to ssDNA ends is impaired and interhomolog recombination is reduced by the presence of DNA:RNA hybrids [11,17]. Interestingly, experiments mapping the genomic locations of DNA:RNA hybrids in *rnh1∆ rnh201∆* diploids showed that hybrids also accumulate during premeiotic S phase at places where there are head-on, but not co-directional, collisions between replication and transcription (called TRCs for Transcription Replication Conflicts) [31].

Given that RNase H-mediated degradation of DNA:RNA hybrids promotes meiotic DSB repair, is the same true for Senataxin/Sen1 helicase activity? *SETX*, the gene that encodes Senataxin, is required for proper mammalian meiosis, as *SETX*^*-/-*^ mutants exhibit male infertility, as well as defects in DSB repair and crossover formation, accumulation of DNA:RNA hybrids and increased apoptosis during prophase I [33,34]. In addition, *SETX* is required for meiotic sex chromosome inactivation (MSCI) [33,35]. This work studies the function of *SEN1* during budding yeast meiosis where meiotic analyses are much easier to perform compared to mammalian cells and the tools and knowledge base are highly sophisticated, allowing more mechanistic experiments to be performed [36].

Meiosis in yeast occurs in the context of a larger process called sporulation, which results in the packaging of the four haploid genomes into gametes called spores [37]. Diploid cells are induced to sporulate when they are starved for nitrogen in the presence of a nonfermentable carbon source. These conditions result in the expression of Ime1 which, in a complex with Ume6, activates the transcription of a set of “early” meiosis-specific genes [38–42]. The early genes include those necessary for premeiotic DNA synthesis, meiotic recombination and chromosome synapsis.

Recombination occurs when the 5’ ends of Spo11-generated DSBs are resected, creating 3’ ssDNA tails to which the Rad51 and Dmc1 recombinases bind [43–45]. The resulting nucleoprotein filaments search the genome for homology and preferentially mediate strand invasion of homologs to form intermediates that are then processed to become either crossovers or noncrossovers [46,47]. An intermediate step in the crossover recombination pathway triggers synapsis, the process whereby a tripartite structure called the synaptonemal complex (SC) assembles between homologous chromosomes [36]. Crossovers, in combination with sister chromatid cohesion, physically connect homologs, allowing them to segregate properly at Meiosis I.

The repair of DSBs in yeast is monitored by the meiotic recombination checkpoint (MRC) [48,49]. The meiosis-specific kinase Mek1 is activated by DSBs [50,51]. Mek1 inhibits the meiosis-specific transcription factor Ndt80 by phosphorylating its DNA binding domain [52]. As DSBs are repaired, Mek1 activity is decreased, allowing Ndt80 to bind the promoters of its target genes and activate transcription of the “middle genes” such as *CDC5*, the polo-like kinase that enables the completion of recombination and exit from prophase I, as well as genes required for Meiosis I and II and spore formation [37,53,54].

This work demonstrates that *SEN1* facilitates three temporally distinct processes during sporulation. First, *SEN1* promotes the timely expression of Ime1-regulated early genes to allow entry into meiosis. Second, *SEN1* prevents the accumulation of DNA:RNA hybrids during premeiotic S phase. Third, during prophase I, *SEN*1 promotes the repair of Spo11-generated DSBs and is required for proper chromosome synapsis.

## Results

### *SEN1* promotes meiotic progression, sporulation and spore viability in yeast

**Fig 1:**
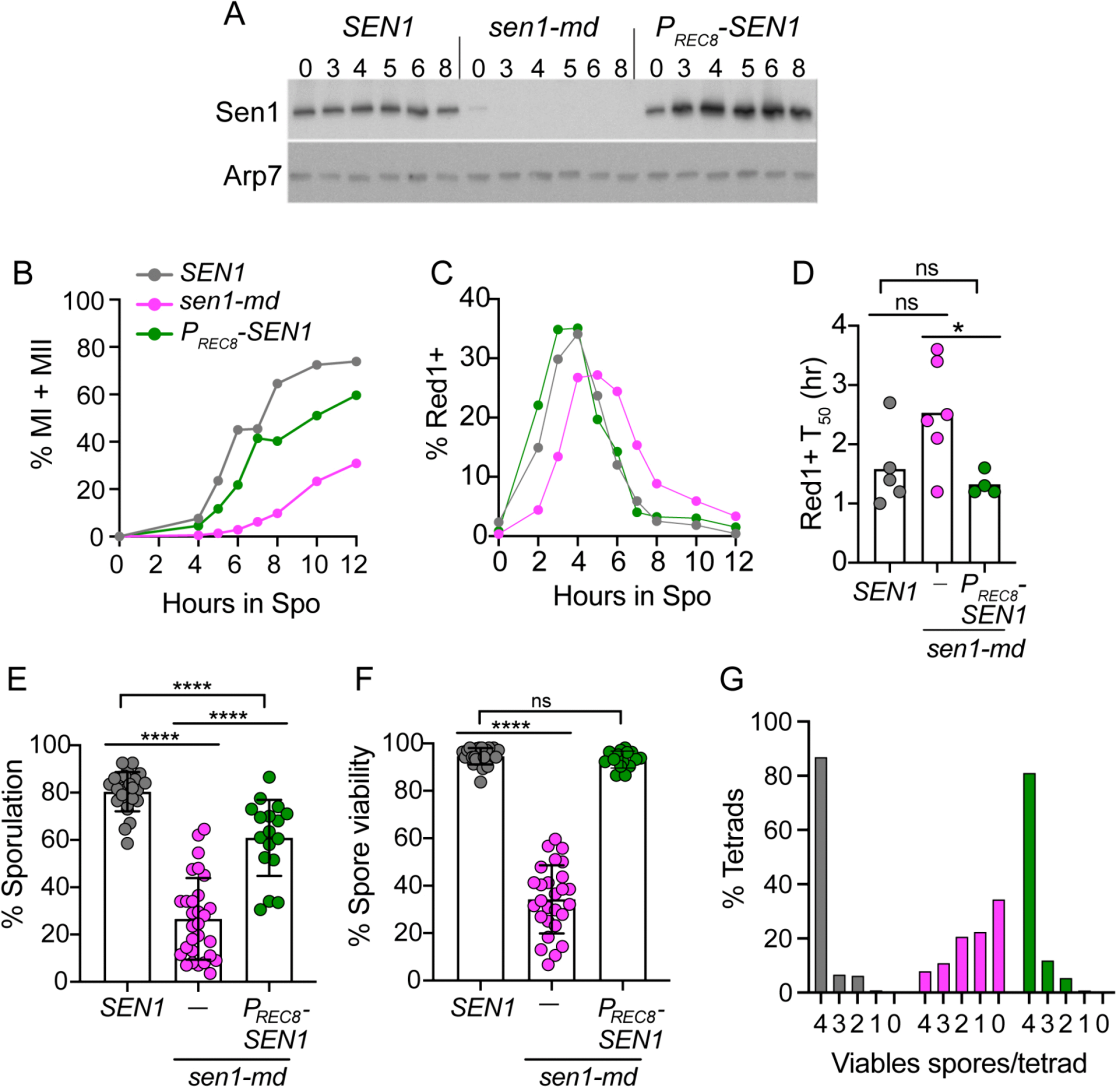
Meiotic depletion of Sen1 results in a prophase I delay and reduces sporulation and spore viability. **(A)** Protein extracts from the indicated meiotic timepoints were generated from *SEN1* (NH716), *sen1-md* (NH2667) and *P*_*REC8*_*-SEN1* (NH2667::pNH410) diploids and probed on immunoblots with either α-Sen1 or α-Arp7 antibodies (Arp7 was used as a loading control). The white line indicates two different blots containing the same samples probed with the indicated antibodies. **(B)** Meiotic progression was followed by staining fixed cells at the indicated timepoints with DAPI and determining the frequency of MI (binucleate) and MII (tetranucleate) cells by fluorescence microscopy. 200 cells were counted at each timepoint for each replicate. *SEN1* (*n *= 7); *sen1-md* (*n* = 8); *P*_*REC8*_*-SEN1* (*n* = 14). The averages of the biological replicates for each strain were plotted. **(C)** Whole cell Red1 immunofluorescence. To determine the frequency of cells in prophase I, whole cell immunofluorescence using α-Red1 antibodies was performed at the indicated timepoints in *SEN1, sen1-md* and *P*_*REC8*_*-SEN1* diploids. The average frequencies of Red1^+^ cells from six replicates of each strain were plotted. **(D)** T_50_ analysis of Red1^+^ cells. The T_50_ value indicates the time it took after transfer to Spo medium to reach the half-maximal % Red1^+^ value. In some replicates, this calculation could not be done due to the absence of data from the 1 hour time point. Data were analyzed for statistical significance using an unpaired, two-tailed Student’s t-test. * = *p* = 0.0291. **(E)** Sporulation. Cultures derived from different single colonies of the indicated strains were sporulated in liquid Spo medium and the number of asci out of 200 cells was determined by light microscopy. Each dot represents a biological replicate. *SEN1* (*n *= 25); *sen1-md* (*n* = 29); *P*_*REC8*_*-SEN1* (*n* = 17) **(F)** Spore viability. Tetrads from the cultures sporulated in Panel E were dissected to determine the frequency of viable spores. Each dot represents the spore viability of one biological replicate containing 23-28 dissected tetrads. The statistical significance of differences between the strains in Panels E and F was determined using the Mann-Whitney test (**** = *p* < 0.0001). ns = not significant. **(G)** Distribution of viable spores in tetrads. The total number of tetrads for each strain was *SEN1* (623), *sen1-md* (728) and *P*_*REC8*_*-SEN1* (395).

Because *SEN1* is essential, a meiotic depletion allele (*sen1-md*) was created by placing *SEN1* under transcriptional control of the *CLB2* promoter, which is not active during meiosis [55,56]. Sen1 protein levels were monitored using a newly developed α-Sen1 antibody. Protein extracts were generated from *SEN1* cells at different times after transfer to sporulation (Spo) medium and probed with α-Sen1 antibodies. A single band corresponding to Sen1’s predicted molecular weight of 253 kD was observed and Sen1 protein levels were constant throughout meiosis (Figs 1A and S1A). In contrast, the amount of Sen1 protein in vegetative cells at 0 hour in the *sen1-md* diploid was reduced relative to *SEN1* and decreased below the limit of detection by 3 hours in Spo medium (Fig 1A).

To validate the antibody, *SEN1* and *sen1-∆N* (an allele that encodes an N-terminal truncation that lacks the first 1003 amino acids out of 2231) were placed under the control of the meiosis-specific *REC8* promoter and transformed into a *sen1-md* diploid [57]. (Throughout the text, *sen1-md*::*P*_*REC8*_*-SEN1* strains are referred to simply as *P*_*REC8*_*-SEN1*). The peptide used to generate the Sen1 antibody contains amino acids 2002–2019 and therefore the antibody should recognize both the full length and truncated Sen1 proteins that exhibit different mobilities on an immunoblot. In the *P*_*REC8*_*-SEN1* diploid, Sen1 was induced by 4 hours in Spo medium compared to vegetative cells (0 hours) (Figs 1A and S1A). In the extracts from the *P*_*REC8*_*-sen1-∆N* diploid, the ~ 250 kD band was replaced by a less abundant protein of ~150 kD, which is close to the expected molecular weight predicted for Sen1^1004-2231^ (135 kD), confirming the antibody’s specificity for Sen1 (S1A Fig).

Phenotypic characterization of the *sen1-md* diploid revealed several meiotic defects. Meiotic progression was examined by staining the DNA with 4’,6-diamidino-2-phenylindole (DAPI) and counting the number of nuclei per cell. Binucleate cells indicate completion of Meiosis I (MI), while tetranucleate cells indicate completion of Meiosis II (MII) (S2 Fig). On average, < 30% of *sen1-md* cells progressed past MI, and those that did were delayed relative to *SEN1* (Fig 1B). In addition, both sporulation and spore viability were significantly reduced in the *sen1-md* diploid (Fig 1E and 1F).

Mutants that decrease interhomolog crossovers exhibit higher levels of inviable spores due to MI non-disjunction. As a result, the number of tetrads in which all four spores are viable goes down, with a coordinated increase in the number of tetrads with two and zero viable spores [58]. This pattern was not observed for *sen1-md* (Fig 1G). While the number of tetrads with 4 viable spores (4:0) was greatly reduced, all other tetrad classes (3:1, 2:2, 1:3, 0:4) were increased, indicating that *sen1-md* spore lethality is unlikely due to MI chromosome mis-segregation (Fig 1G).

While the reduced amount of Sen1 protein present in vegetative *sen1-md* cells is sufficient for viability, it may not be sufficient for other *SEN1* functions (Fig 1A). This fact raises the possibility that the meiotic progression delay, reduced sporulation and decreased spore viability phenotypes result from DNA damage incurred during vegetative growth, rather than defects in meiotic processes. To test this idea, a *P*_*REC8*_*-SEN1* strain was examined to see if expressing *SEN1* after the initiation of meiosis can complement *sen1-md* meiotic phenotypes. In fact, *P*_*REC8*_*-SEN1* partially rescued the meiotic progression delay and sporulation defect (Fig 1B and 1E) and restored spore viability to *SEN1* levels (Fig 1F and 1G). Thus, meiosis-specific expression of *SEN1* is sufficient to reverse the meiotic defects of the *sen1-md* mutant, suggesting a requirement for Sen1 activity during normal meiosis.

A delay in meiotic progression was previously observed in the absence of RNase H activity (*rnh1∆ rnh201∆*) where it is known that DNA:RNA hybrids form on the single- stranded tails of DSBs, thereby making them difficult to repair [17,31]. The MRC delays exit from prophase I when a threshold number of DSBs is present. If the meiotic progression delay in *sen1-md* is also due to impaired meiotic DSB repair, then cells should take longer to exit prophase I. Prophase I cells can be distinguished using whole cell immunofluorescence with antibodies that detect Red1, a meiosis-specific axial element protein that is degraded upon exit from pachynema [54,59,60]. This analysis found that both entry into and exit from prophase I were affected in the *sen1-md* diploid, indicating there are two separate components to the meiotic progression delay. First, the appearance of Red1^+^ cells in *sen1-md* was delayed by ~2 hours compared to *SEN1*, indicating that *SEN1* is necessary for efficient entry into meiosis (Fig 1C). Second, the *sen1-md* downward slope due to exit from prophase I occurred later in *sen1-md* relative to *SEN1* (Fig 1C). A diploid ectopically expressing *SEN1* using *P*_*REC8*_*-SEN1* exhibited similar prophase I kinetics as *SEN1*, indicating that the MRC was no longer being triggered (Fig 1C and 1D). These results indicate that *SEN1* is also required for timely progression out of prophase I.

### *SEN1* promotes expression of *IME1* regulated genes

*REC8* is an early meiotic gene whose transcription is activated by Ime1 [38,39,42,61]. One explanation for why *P*_*REC8*_*-SEN1* only partially rescues the *sen1-md* meiotic progression delay is that *SEN1* promotes the timely transcription of genes regulated by Ime1. As a first test of this hypothesis, the timing of expression for several early meiosis-specific proteins was examined.

*HOP1*, *RED1* and *REC8* encode meiosis-specific components of the axial elements formed along sister chromatids [59,61–63]. *MEK1/MRE4* encodes a meiosis-specific kinase that regulates many different steps of meiotic recombination, as well as the MRC [52,64–70]. Hed1 is a meiosis-specific protein that binds to Rad51 to inhibit its strand exchange activity during prophase I [71,72]. Mek1 phosphorylation of Hed1 threonine 40 stabilizes the protein and can be used as a marker for Mek1 kinase activity [53,73].

In the *SEN1* diploid, Rec8 protein was first observed after 2 hours in Spo medium, its level peaked at 4 hours and was nearly undetectable by 8 hours (S1B Fig). By 4 hours, most of the cells were in prophase I, as indicated by peak levels of Red1 and meiotic DSBs [indirectly indicated by Hop1 phosphorylation (pHop1)] (Figs 1C and S1B) [74]. Mek1 activity, indicated by phosphorylated Hed1 (pHed1), also peaked at 4 hours, in keeping with the larger amount of phosphorylated Hop1 (S1B Fig) [73]. Red1 was nearly gone by 6 hours consistent with cells progressing into MI (Figs 1B, 1C and S1B). In contrast, all 5 proteins in the *sen1-md* diploid peaked later than 4 hours and were still present at 10 hours, consistent with a portion of the cells being delayed in prophase I. Notably, the Rec8 protein from the *P*_*REC8*_*-SEN1* diploid exhibited similar expression kinetics as in *sen1-md* (S1B Fig).

**Fig 2:**
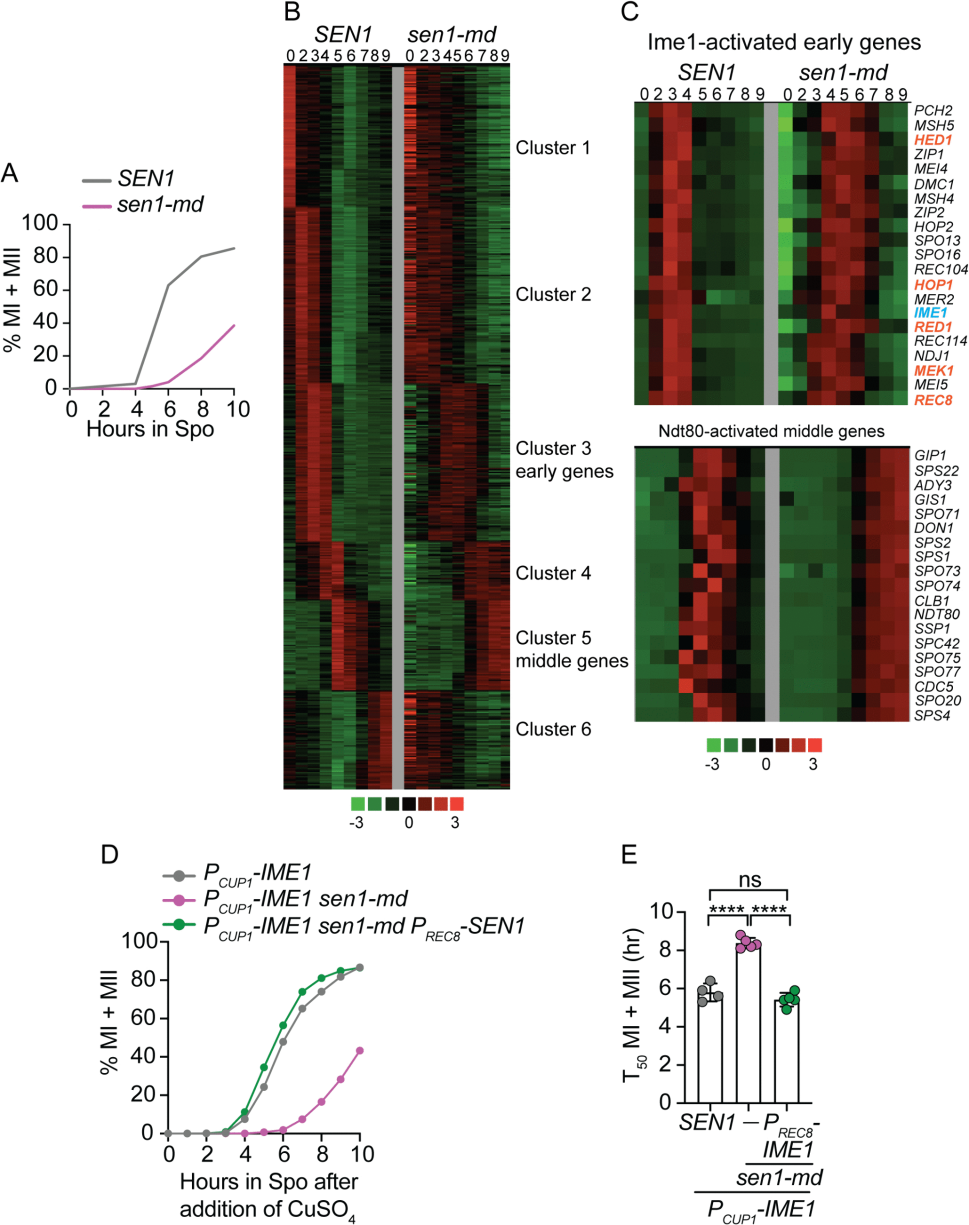
*SEN1* specifically regulates *IME1* expression during meiosis. *SEN1* (NH2473) and *sen1-md* (NH2520) were transferred to Spo medium and cells were taken at various timepoints. **(A)** Meiotic progression. Fixed cells were stained with DAPI and examined by fluorescence microscopy for the presence of MI and MII cells. **(B)** RNA-seq analysis. mRNA was isolated at the indicated timepoints from the timecourse shown in Panel A. Numbers above each column indicate hours after transfer to Spo medium. The *z* value for each timepoint (*n*) was calculated as [(FPKM_*n*_-FPKM_ave_)/SD], where FPKM = fragments per kilobase per million, SD = standard deviation and ave = average. Genes were sorted based on the RNA expression patterns in the *SEN1* diploid by clustering analysis (k-means) using Cluster 3.0. The heat map was generated using Java TreeView. Green indicates *z* values < 0, while red indicates *z* values > 0. **(C)** A subset of previously identified *IME1*-dependent early genes and *NDT80*-dependent middle genes were selected and clustered as in Panel B. Gene names in orange encode proteins that were examined in S1B Fig, while *IME1* is written in blue. **(D)** Meiotic progression in *P*_*CUP1*_*-IME1* diploids. Diploids containing *P*_*CUP1*_*-IME1* (39807; *n *= 4), *P*_*CUP1*_*-IME1 sen1-md* (NH2842; *n *= 5) or *P*_*CUP1*_*-IME1 sen1-md::P*_*REC8*_*-SEN1* (NH2842::pBG45; *n *= 5) were incubated in Spo medium for 2 hours at which time CuSO_4_ was added to a final concentration of 50 μM to induce *IME1* expression. Cells at the indicated timepoints were stained with DAPI and meiotic progression analyzed as in Fig 1B. The 0 hour timepoint is the time of copper addition. The average values for each timepoint were graphed. **(E)** T_50_ analysis of the timecourses from Panel D. The T_50_ value indicates the time it took after the addition of copper at which the half-maximal %MI + MII value was reached. Data were analyzed for statistical significance using an unpaired, two-tailed Student’s t-test. **** = p < 0.0001.

To more directly test the hypothesis that *SEN1* promotes early gene transcription, RNA sequencing analysis was performed using cells taken at different times after transfer to Spo medium. As was previously observed, meiotic progression in the *sen1-md* diploid was delayed in this time course relative to *SEN1* (Fig 2A). Genes were clustered by k-means based on RNA expression patterns in the *SEN1* diploid. The heat map for the *SEN1* strain exhibited gene clusters that agreed well with the literature (Fig 2B) [38,42]. When a subset of early genes from the *SEN1* diploid known to function in prophase I was examined, their expression nearly uniformly peaked at 3–4 hours after transfer to Spo medium (Fig 2C). In contrast, expression of the same set of genes in the *sen1-md* mutant was delayed, with broader peaks occurring between 4 and 6 hours. The delay in early gene transcription correlated with the later appearance of *IME1* transcripts, the master regulator for early gene transcription, in the *sen1-md* strain compared to *SEN1* (Fig 2C). As expected, the delay in early gene transcription coordinately delayed the expression of *NDT80*-regulated middle genes (Fig 2B and 2C). We propose that meiotic progression proceeds more slowly in *sen1-md* for two reasons: (1) a delay in the onset of early gene transcription, and (2) activation of the MRC due to the presence of unrepaired DSBs. This hypothesis would explain the partial rescue of the meiotic progression defect in *P*_*REC8*_*-SEN1*. Ectopic expression of *SEN1* during meiosis using *P*_*REC8*_*-SEN1* would allow DSB repair, thereby deactivating the MRC and allowing cells to progress faster than *sen1-md*. However, because *SEN1* transcripts produced using the Ime1-regulated *REC8* promoter appeared too late to fix the early gene transcription defect, the cells still exhibited a delay in progression through the early stages of meiosis.

To test whether *SEN1* is involved specifically in the regulation of *IME1* expression, the *sen1-md* mutant was introduced into a strain in which *IME1* was under the control of the copper-inducible *CUP1* promoter [75]. If *SEN1* regulation of the *IME1* promoter is responsible for the early gene transcriptional delay, then *P*_*CUP1*_*-IME1* should eliminate this component of the delay. The *P*_*CUP1*_*-IME1 sen1-md* diploid exhibited delayed progression compared to *P*_*CUP1*_*-IME1* after the addition of copper, as expected if the MRC was being triggered by unrepaired prophase I DSBs (Fig 2D). However, when *P*_*REC8*_*-SEN1* was present in the *P*_*CUP1*_*-IME1 sen1-md* background, the kinetics of meiotic progression was the same as *P*_*CUP1*_*-IME1* (Fig 2D and 2E). These results confirm the hypothesis that *SEN1* specifically promotes *IME1* expression to allow entry into meiosis.

### *SEN1* suppresses aberrant transcription of regulatory sequences upstream of *IME1*

**Fig 3:**
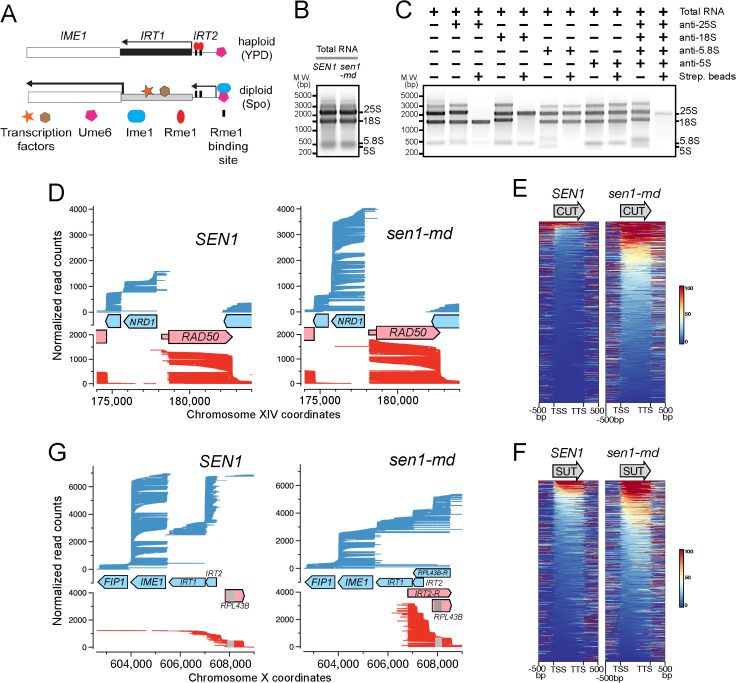
Direct RNA sequencing in *SEN1* and *sen1-md* diploids after 2 hours in Spo medium. **(A)** Schematic model of the *IME1* region. In haploid cells, Rme1 activates transcription of the noncoding *IRT1* RNA that inhibits transcription of *IME1* by creating a “repressive chromatin state” (black bar). In diploid cells in Spo medium, *RME1* expression is repressed and glucose repression is removed, lowering the amount of *IRT1*, thereby allowing *IME1* transcription. Ime1 then binds to Ume6 upstream of *IRT2* and activates transcription of this non-coding RNA. **(B)** Total RNA extracted from *SEN1* diploid cells after 2 hours in Spo medium was fractionated on a 1% agarose gel in 1xTAE containing 1% bleach and stained with SYBR Gold. **(C)** Total RNA was incubated with the indicated biotinylated antisense RNAs targeting specific rRNAs, followed by precipitation using streptavidin-coated magnetic beads. **(D)** Individual RNA molecules detected by dRNA-seq from *SEN1* and *sen1-md* were sorted by their 3’-end positions and subsequently by their 5’*-*end positions before plotting along the indicated chromosomal region. The step size of the read counts in *sen1-md* was set to 1.2 times that of *SEN1* to normalize for the number of uniquely mapped reads for the combined replicates (12,637,870 and 15,252,836, respectively). Red lines: Watson strand. Blue lines: Crick strand. **(E)** Heatmaps of read coverage (normalized to count per million) for *SEN1* and *sen1-md* were plotted across 918 annotated CUT regions. Each region was scaled to equal length and aligned at its annotated transcription start (TSS) and termination sites (TTS), with 500 bp of flanking sequence. Data were sorted according to transcript abundance within the CUT regions. **(F)** Same as E, except that 833 SUT regions were plotted. **(G)** Same as D, except that the region encompassing *IME1* was plotted. Grey lines: intronic regions. The tests for statistical significance for conclusions based on Panels D and G are described in the Methods.

The regulation of *IME1* expression is complex, as it requires integration of signals relating to both mating type and nutrients (reviewed in [76,77]. The *IME1* promoter is approximately 2 kb in length [78,79]. Within the first 1.1 kb are multiple regulatory sites, including ones that repress *IME1* transcription when glucose is present or activate transcription when cells are exposed to acetate [79]. In haploid cells, transcription of a long noncoding RNA called *IRT1* beginning 1.4 kb upstream of the *IME1* gene creates a repressive chromatin state that inhibits *IME1* transcription (Fig 3A) [80]. *IRT1* transcription is activated by a repressor of meiosis, *RME1*, a transcription factor that binds to two sites upstream of *IRT1* [80,81]. Transcription of *RME1* itself is repressed in diploids by the a1/α2 repressor encoded by *MAT***a** and *MAT*α, thereby preventing transcription of *IRT1* and allowing expression of *IME1* when diploid cells are in Spo medium (Fig 3A) [81]. In case the repression of *RME1* expression is leaky, transcription of a second long non-coding RNA called *IRT2*, located immediately upstream of *IRT1*, prevents Rme1 from binding to its sites in the *IME1* promoter, thereby ensuring that *IRT1* transcription is low (Fig 3A) [82]. *IRT2* transcription is activated by a complex formed between Ume6 and Ime1 [82].

To investigate how *SEN1* contributes to the regulation of *IME1*, nanopore direct RNA sequencing (dRNA-seq) was performed on coding and non-coding transcripts isolated from *SEN1* and *sen1-md* cells in Spo medium [83]. dRNA-seq requires that each transcript has a 3’ poly(A) tail to which a sequencing adapter and motor protein that threads the RNA through a pore can be ligated. Non-coding RNAs, particularly those that fail to terminate properly in the absence of the Nrd1-Nab3-Sen1 (NNS) complex, lack polyA tails and therefore would be underrepresented [84]. To capture these transcripts, poly(A) tails need to be added *in vitro*. However the high abundance of the four ribosomal RNAs (rRNAs) (25S, 18S, 5.8S and 5S) in total RNA preparations would overwhelm this reaction and so must be removed prior to polyadenylation (Fig 3B). The four rRNAs were depleted from total RNA isolated from *SEN1* and *sen1-md* cells by hybridization to the combined biotinylated antisense transcripts for each rRNA, followed by streptavidin-based removal (Fig 3C). The resulting rRNA-depleted RNA was then polyadenylated *in vitro* using *E. coli* poly(A) polymerase prior to nanopore library preparation.

Two biological replicates were performed for *SEN1* and *sen1-md* strains after two hours in Spo medium, yielding ~6–7 million uniquely aligned reads per replicate to the *S. cerevisiae* SK1 genome. To assess data quality, sequencing reads mapped within 5,496 annotated transcript regions were quantified using featureCounts, followed by normalization and analysis with DESeq2 [85–87]. Strong correlations (*r* > 0.95) were observed between biological replicates of the same genotype, whereas lower correlations between *SEN1* and *sen1-md* samples highlight condition-specific transcriptional changes (S3A Fig). In dRNA-seq, reads initiate at the poly(A)-anchored 3’ end and proceed in a 3’-to-5’ direction; therefore RNA fragmentation or degradation can yield 5’-truncated reads and under-representation of 5’ ends. As a result, transcripts sharing the same 3’ end frequently have heterogeneous 5’ ends. This is a known limitation of dRNA-seq on the Oxford Nanopore platform [83].

In wild-type cells, *SEN1* negatively regulates the activity of the NNS complex by promoting premature termination of *NRD1* transcription [88]. Consistent with this role, *NRD1* transcript levels in the *sen1-md* mutant were 4.6-fold higher than in *SEN1* (Figs 3D and S3B). No significant difference in transcript levels was observed for the adjacent *RAD50* gene, although the transcription initiation site was altered to give a longer 5’ untranslated region in *sen1-md* (Figs 3D and S3B). The NNS complex is also required to suppress the expression of non-coding RNAs by coupling *SEN1-*dependent termination to TRAMP-mediated exosome degradation [22]. Consistent with this fact, *sen1-md* exhibited an accumulation of cryptic unstable transcripts (CUTs) and, to a lesser extent, stable unannotated transcriptions (SUTs) (Fig 3E and 3F) [89–91]. In addition, while the amount of sense transcripts for protein coding genes was similar between *SEN1* and *sen1-md*, antisense transcripts were modestly elevated in *sen1-md* (S3D Fig). These results indicate that there were widespread termination defects caused by impaired Sen1 function.

The *SEN1* diploid exhibited the expected transcript pattern in the *IME1* region, i.e., a high level of *IME1* and *IRT2* RNAs and a lower level of *IRT1* RNA (Fig 3G) [82]. In addition, the *RPL43B* gene immediately upstream of *IRT2* was transcribed in the opposite orientation as expected. This pattern was significantly altered in *sen1-md*. First, *IME1* transcripts were significantly reduced in *sen1-md* relative to *SEN1* which explains why it takes longer for *sen1-md* cells to activate transcription of the early genes and enter meiosis (Figs 3G and S3B). Second, novel transcripts originating within *IRT1* with opposite polarity spanned the *IRT2* sequence (Fig 3G). The genomic location of these antisense transcripts was annotated as *IRT2-R*. In addition, a high level of antisense transcripts spanning *RPL43B* was also observed (annotated *RPL43B-R*) (Fig 3G). These results demonstrate that *SEN1* suppresses the accumulation of antisense transcripts upstream of *IME1* that may interfere with *IME1* transcription.

### The *sen1-md* delay in exiting prophase I is due to the meiotic recombination checkpoint

The MRC prevents cells from exiting prophase I when DSB levels are high because activated Mek1 inhibits Ndt80-dependent transcription [52,60,92,93]. Mek1 interacts with Ndt80 through a five-amino acid sequence in the transcription factor and deletion of this sequence abrogates the MRC [52]. The *NDT80* allele containing this five-codon deletion is referred to as *NDT80-mid* (Mek1 Interaction Defective). *NDT80-mid* specifically disrupts the MRC without affecting Mek1 kinase activity and cells progress through meiosis with the same kinetics as *NDT80* strains [60]. Therefore *NDT80-mid* is a useful tool for determining whether the MRC is triggered by a particular mutant.

**Fig 4:**
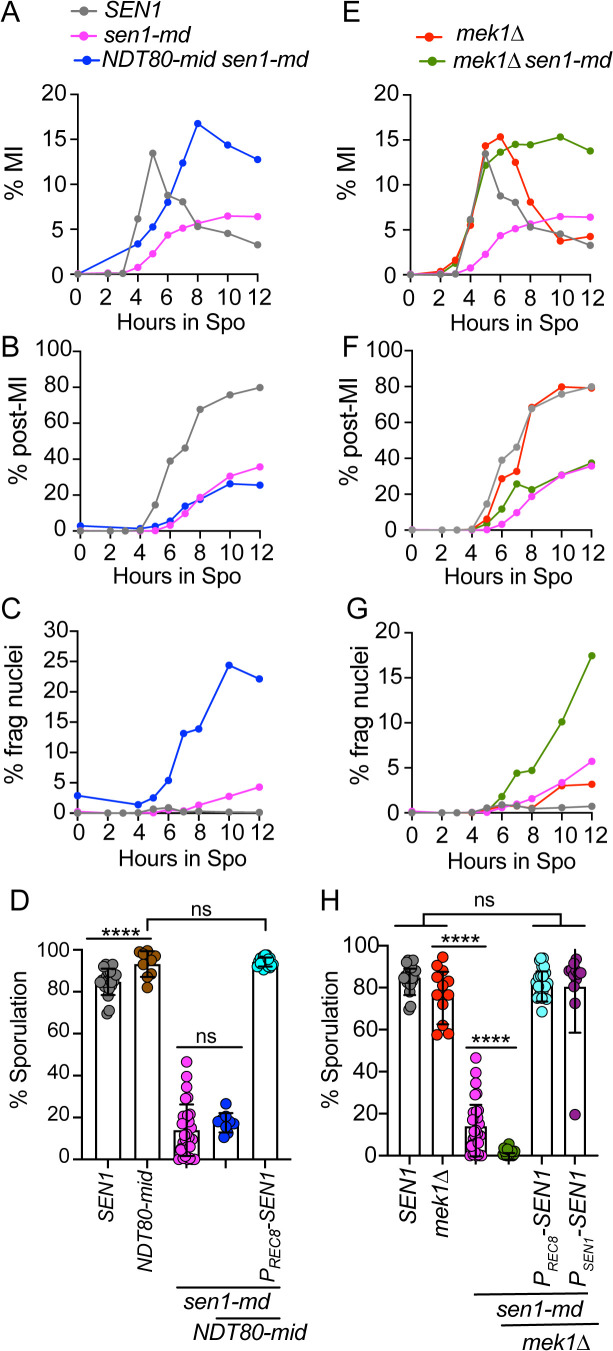
Meiotic progression and sporulation of *sen1-md* diploids in the absence of the meiotic recombination checkpoint. *SEN1* (*n* = 13), *sen1-md* (*n* = 20), *NDT80-mid sen1-md* (NH2667::pNH317^2^) (*n* = 4), *mek1∆* (NH729) (*n* = 6), and *mek1∆ sen1-md* (NH2669) (*n* = 10) diploids were induced to undergo meiosis and the average frequency of the indicated cell types determined at various time points as described in Fig 1B. **(A, E)** binucleate cells that have completed MI; **(B, F)** Post-MI includes both MII cells (tetranucleate) and cells with fragmented nuclei (>4 DAPI foci). **(C, G)** Cells with fragmented nuclei. **(D, H)** Different colonies from the strains used in Panels A and E, as well as *NDT80-mid P*_*REC8*_*-SEN1* (NH2667::pNH410::pNH317^2^), *mek1∆ P*_*REC8*_*-SEN1* (NH2669::pNH410), and *mek1∆ P*_*SEN1*_*-SEN1* (NH2669::pBG28), were sporulated at 30°C on solid medium. Each dot represents a biological replicate for which the percentage of sporulated cells was determined for 200 cells. The *SEN1* and *sen1-md* data are repeated in A-H. Error bars indicate the means and standard deviations. Statistical significance of differences between strains in D and H was determined using the Mann-Whitney test (* = *p* < 0.02; ** = *p* < 0.002; *** = *p* < 0.001; **** = *p* < 0.0001).

In the *SEN1* strain, MI cells peaked at ~13% after only 5 hours in Spo medium (Fig 4A). In contrast only ~7% of the *sen1-md* cells had completed MI after 12 hours (Fig 4A). The onset of MI occurred later than *SEN1* in both *NDT80-mid sen1-md* and *sen1-md* due to the delay in early gene transcription. However, *NDT80-mid sen1-md* cells completed MI faster than *sen1-md*, peaking with ~18% cells at 8 hours (Fig 4A). The fact that the MRC is responsible for the prophase I arrest/delay observed when Sen1 is meiotically depleted indicates that *SEN1* is required for timely DSB repair.

Although *NDT80-mid* allowed some *sen1-md* cells to exit prophase I, it did not rescue the sporulation defect (Fig 4D). This may be because allowing cells to proceed through the meiotic divisions with unrepaired DSBs resulted in fragmented nuclei, as indicated by the presence of cells with more than 4 DAPI staining foci, similar to what is observed when the MRC is inactivated in *dmc1∆* or *sae2∆* diploids (S2 Fig) [92,94]. Cells containing either four nuclei (i.e., completed MII) or fragmented nuclei were classified as “post-MI”. Greater than 80% of *SEN1* cells were post-MI by 12 hours with <5% showing fragmented nuclei (Fig 4B and 4C). In contrast, only ~25% of *sen1-md* and *NDT80-mid sen1-md* cells proceeded past MI (Fig 4B). A major difference between these two strains, however, was that almost all the *NDT80-mid sen1-md* post-MI cells contained fragmented nuclei, compared to <5% of *sen1-md* cells (Fig 4C). Therefore, the prophase I delay created by the MRC in the *sen1-md* diploid provided time for some DSBs to be repaired.

*MEK1* controls not only the MRC, but also interhomolog bias and inhibition of Rad51 strand exchange activity [67,69,73]. For example, when *MEK1* is inactive in a *dmc1∆* mutant, cells progress through meiosis with intact chromosomes because Rad51 repairs DSBs using sister chromatids, thereby removing the signal that activates the MRC [49,74]. In contrast, if DSBs are unrepairable using either homologs or sister chromatids as templates (e.g., *sae2∆* or *rad50S*), cells progress through meiosis with fragmented nuclei in the absence of the MRC [49,94]. Similar to *NDT80-mid*, combining *mek1∆* with *sen1-md* resulted in faster entry into MI compared to *sen1-md* with a reduced number of post-MI cells (Fig 4E and 4F). The increased frequency of fragmented nuclei in the *mek1∆ sen1-md* diploid suggests that DSBs persisted because they were unrepairable (Fig 4G). The sporulation defects of *NDT80-mid sen1-md* and *mek1∆ sen1-md* were rescued by *P*_*REC8*_*-SEN1* to the same extent as the constitutively expressed *P*_*SEN1*_*-SEN1*, consistent with a need for *SEN1* during prophase I to allow DSB repair (Fig 4D and 4H).

### *SEN1* plays a minor role in crossover formation at the *HIS4LEU2* DSB hotspot

**Fig 5:**
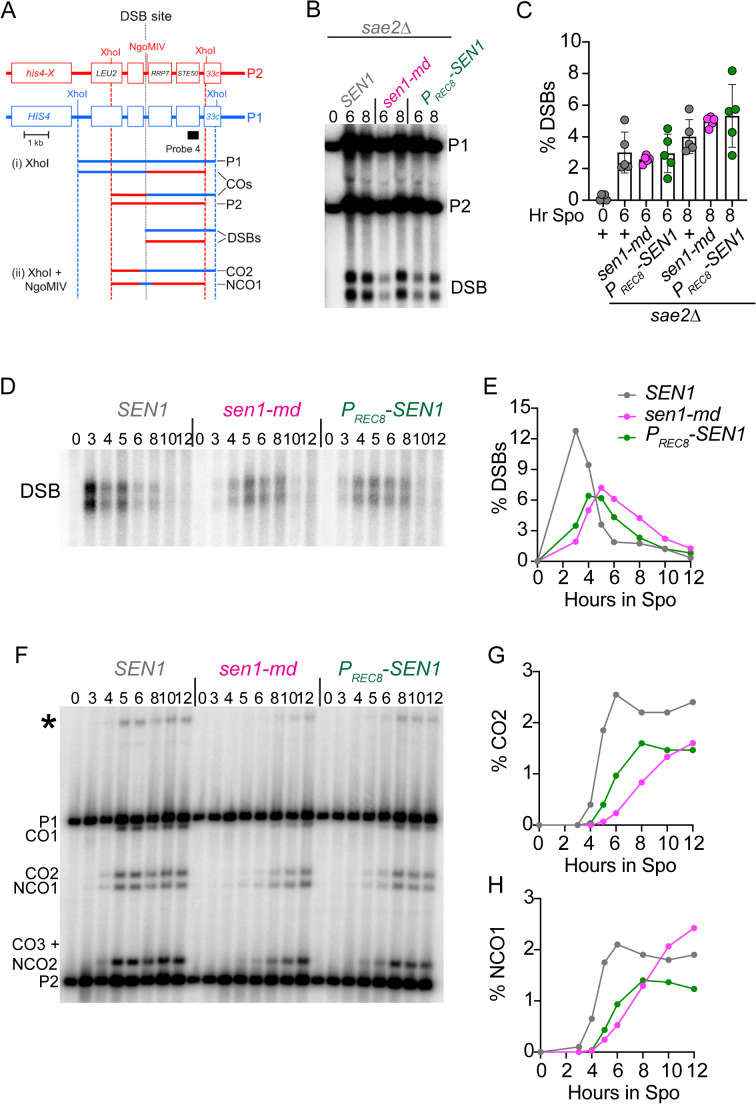
Physical analysis of DSBs, crossover and noncrossover formation during meiosis in *sen1-md* and *P*_*REC8*_*-SEN1* diploids. **(A)** Schematic of the *HIS4LEU2* hotspot (taken from [64]). The homologs, P1 and P2, have XhoI sites located at asymmetric positions. The dotted vertical line indicates where Spo11 makes DSBs. The NgoMIV site is adjacent to the DSB site on P1. The black box indicates the sequence used as a probe in Southern blot analysis. **(B)** DSBs in *sae2∆* diploids. DNA was isolated from *sae2∆* (NH1054), *sae2∆ sen1-md* (NH2846), and *sae2∆ sen1-md::P*_*REC8*_*-SEN1* (NH2846::pNH410)(*n* = 5 for all) at the indicated timepoints and digested with XhoI to detect DSBs. The XhoI sites on the two homologs are slightly offset, resulting in two DSB fragments. One replicate is shown. **(C)** Quantification of the total DSBs from the experiment in B. The amount of DNA in the two DSB fragments was combined and divided by the total amount of DNA for the five replicates in each strain at each timepoint. None of the pairwise combinations at either 6 or 8 hours were significantly different based on an unpaired, two-tailed Student’s T test. **(D)** Timecourses of *SEN1* (*n* = 2), *sen1-md* (*n* = 3) and *P*_*REC8*_*-SEN1* (*n* = 3) diploids were performed and genomic DNA isolated from cells at the indicated timepoints. Genomic DNA was digested with XhoI to detect Spo11-generated breaks. The entire gel for the representative replicate shown here is presented in S4 Fig. **(E)** Average DSB values for the different replicates normalized to the total amount of DNA. **(F)** CO and NCO: Genomic DNA was digested with XhoI and NgoMIV. CO1 and CO2 indicate crossover bands, while NCO1 and NCO2 represent noncrossover bands. (G) average %CO2 values. (H) average %NCO1 values. The asterisk indicates a band containing a flanking XhoI site gene conversion.

To determine directly whether *SEN1* promotes meiotic recombination, physical analyses using the *HIS4LEU2* hotspot were performed. This well characterized hotspot has asymmetric XhoI sites that give rise to distinct bands corresponding to DSBs and crossovers (COs) that can be detected using Southern blots (Fig 5A) [95]. In addition, there is an NgoMIV site on one homolog adjacent to the region where Spo11 makes DSBs. Digestion with both XhoI and NgoMIV therefore also allows detection of noncrossovers (NCOs) resulting from gene conversion events involving the NgoMIV site [96].

A reduction in recombination products will be observed if there is a defect in DSB formation. Whether *SEN1* promotes DSB formation was tested using an *sae2∆/com1∆* mutant, in which DSBs are generated, but Spo11 remains covalently attached to the 5’ ends, thereby preventing resection and repair [97,98]. No significant differences were observed for the levels of DSBs in *sae2∆*, *sae2∆ sen1-md* or *sae2∆ P*_*REC8*_*-SEN1* strains after both 6 and 8 hours in Spo medium. *SEN1* therefore does not promote Spo11-mediated DSB formation at the *HIS4LEU2* hotspot (Fig 5B and 5C).

In the *SEN1* diploid, DSBs peaked at 3 hours after transfer to Spo medium and then rapidly disappeared (Fig 5D and 5E) (S4 Fig). In contrast, DSB formation was delayed in both *sen1-md* and *P*_*REC8*_*-SEN1*. This was expected as transcription of most of the genes required for making DSBs (e.g., *SPO11*, *REC114*, *REC102* and *REC104*) is dependent on *IME1*. DSBs also persisted longer in *sen1-md* than in either *SEN1* or *P*_*REC8*_*-SEN1* and were still detectable after 12 hours (Fig 5E). CO2 and NCO1 fragments that can be unambiguously identified on the XhoI/NgoMIV gel were quantified (Fig 5F). In the *SEN1* strain, CO2 formation plateaued by 6 hours. In contrast, CO formation in *sen1-md* was delayed and reduced by 25% at 12 hours but still had not reached a plateau (Fig 5G). The kinetics of CO formation in the *P*_*REC8*_*-SEN1* strain was intermediate between *SEN1* and *sen1-md*, as expected if the *sen1-md* early gene transcription delay was followed by more efficient DSB repair due to the presence of *SEN1*. However, for reasons that are unclear, the level of COs in the *P*_*REC8*_*-SEN1* strain resembled that of *sen1-md* and did not reach *SEN1* levels.

A different phenotype was observed for NCOs in the *sen1-md* diploid. Instead of being decreased, there were more NCOs at 12 hours compared to *SEN1* (Fig 5F and 5H). This result could be because prophase I length is longer in many of the *sen1-md* cells, which delays the expression of the polo-like kinase, *CDC5*, required for resolution of the double Holliday junctions into COs [54]. Furthermore, cells arrested in pachynema continue to make DSBs and produce NCOs by synthesis-dependent strand annealing [99–101]. The idea that the increase in NCO formation in *sen1-md* is due to a delay in prophase I exit is supported by the observation that in the *P*_*REC8*_*-SEN1* diploid that was able to progress, NCOs plateaued by 8 hours (Fig 5F and 5H).

The effects of *sen1-md* on interhomolog recombination are small and therefore their significance is difficult to determine with a limited number of replicates. We therefore repeated the CO and NCO analyses at the 8 and 12 hour timepoints using 5 replicates from the *SEN1*, *sen1-md* and *P*_*REC8*_*-SEN1* diploids. Sporulation and spore viability in the replicates from the three strains were consistent with previous results (S5A and S5B Fig). A slight reduction in the CO2 band was observed between the *SEN1* and *sen1-md* diploids (~20%) at both the 8 and 12 hour timepoints, although only the 8 hour values were statistically significant (S5C and S5D Fig). In contrast, the NCO1 values were similar for *SEN1* and *sen1-md* at both timepoints (Figs 5E and S5C). For reasons that are unclear, the *P*_*REC8*_*-SEN1* COs and NCOs were reduced compared to *sen1-md* in contrast to the previous timecourses. *SEN1* may have a differential effect on DSBs that form crossovers, but this effect is minor.

### *SEN1* promotes chromosome synapsis during meiosis

In budding yeast, meiotic DSB repair by a crossover specific pathway containing a functionally diverse set of “ZMM” proteins is required for SC formation [102–107]. The SC is a tripartite supramolecular chromosomal complex that initiates assembly from discrete chromosome sites associated with recombination (as well as from centromeres) and extends along the full lengths of aligned homologous chromosome axes [108,109]. SC formation in yeast is completely dependent on DSB formation and early processing. Diploids containing *spo11∆*, *sae2∆* or *rad50S* mutants exhibit only aggregates of SC structural proteins (polycomplexes) instead of linear SC structures, while the DSB repair defects of *dmc1∆* or *msh4∆* are associated with an overall decrease and delay in SC formation, rather than a complete abrogation of SC [110–116].

**Fig 6:**
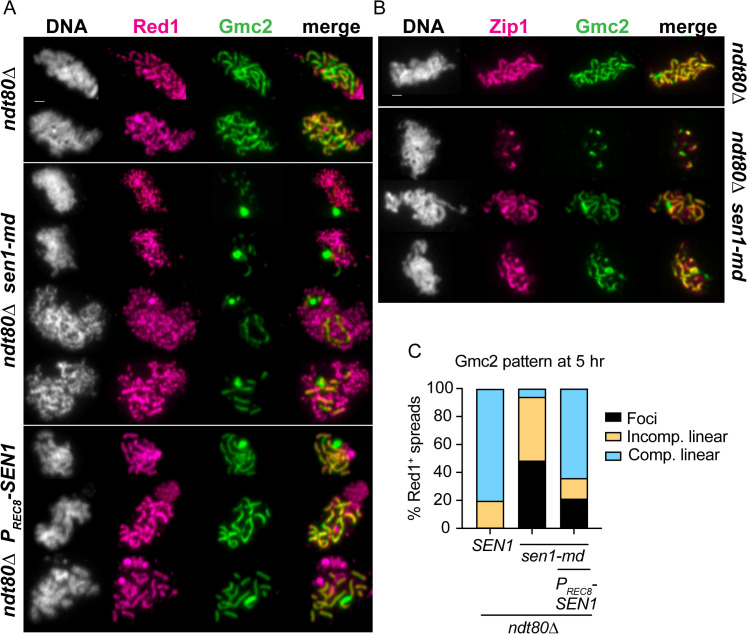
SC assembly in *ndt80∆*, *ndt80∆ sen1-md* and *ndt80∆ P*__*REC8*__*-SEN1* diploids. The *ndt80∆* (NH2188, 156 nuclei), *ndt80∆ sen1-md* (AM6697, 169 nuclei) and *ndt80∆ P*_*REC8*_*-SEN1* (AM6697::pNH410, 75 nuclei) diploids were transferred to Spo medium for 5 hours at which time cells were processed to make chromosome spreads. **(A)** Immunofluorescence on surface-spread meiotic chromosomes to detect chromosome axes (Red1) and the central element (Gmc2). Each row corresponds to a different surface-spread nucleus, with the genotypes indicated on the left. DNA is labeled with DAPI (white, first column), α-Red1 (magenta, second column) or α-Gmc2 (green). The last column shows the merged image. Bar, 1 μm. **(B)** Same as A except that α-Zip1 antibodies were used to detect the central region of the SC. **(C)** Histogram of the fraction of total nuclei at 5 hours with completely synapsed chromosomes (Comp. linear = full linear structures of Gmc2 that extend throughout the full spread nucleus), incomplete SC (incomp. linear = shorter linear structures of Gmc2 that only partially extend throughout the surface-spread nucleus), or Gmc2 foci. Only those surface-spread nuclei with maximal Red1 accumulation on chromosome axes were analyzed.

Diploids homozygous for *ndt80∆* arrest in pachynema, the stage of prophase I when chromosomes are fully synapsed [93]. The ability of *ndt80∆ sen1-md* diploids to assemble SCs was assessed by examining the distribution of SC structural components Gmc2 (part of the SC central element) and Zip1 (an SC transverse filament protein) on surface-spread meiotic chromosomes that displayed maximal Red1 accumulation after 5 hours in Spo medium [117,118]. At this timepoint, 80% of the *ndt80∆* control nuclei exhibited full-length or nearly full-length linear structures of Gmc2 throughout the entirety of the mid-prophase nucleus, while the remaining spread chromosomes showed shorter linear assemblies of Gmc2 (Fig 6A and 6C). By contrast, only 6% of the surface-spread nuclei from the *ndt80∆ sen1-md* strain exhibited full-length linear Gmc2 structures, even though most spreads displayed Red1 accumulation on chromosome axes to the same extent as the *ndt80∆* control. The remaining nuclei were nearly evenly divided between those that had only Gmc2 foci (48%) and those with shorter linear Gmc2 assemblies (46%) (Fig 6A and 6C). In *ndt80∆ sen1-md* cells, the Gmc2 linear assemblies detected on chromatin co-localized with Zip1, indicating that these structures correspond to mature (albeit partial) SCs (Fig 6B). Expression of *SEN1* from the *REC8* promoter partially rescued the *sen1-md* synapsis defect, increasing the frequency of Red1^+^ nuclei with complete SCs from 6% to 64% (Fig 6A and 6C). The partial complementation may be due to variability in the timing of expression by the *REC8* promoter, with *SEN1* being expressed too late in some cells. *SEN1* therefore indirectly plays an important role in promoting chromosome synapsis during yeast meiosis.

### *SEN1* prevents formation of *SPO11*-independent DSBs during meiosis

**Fig 7:**
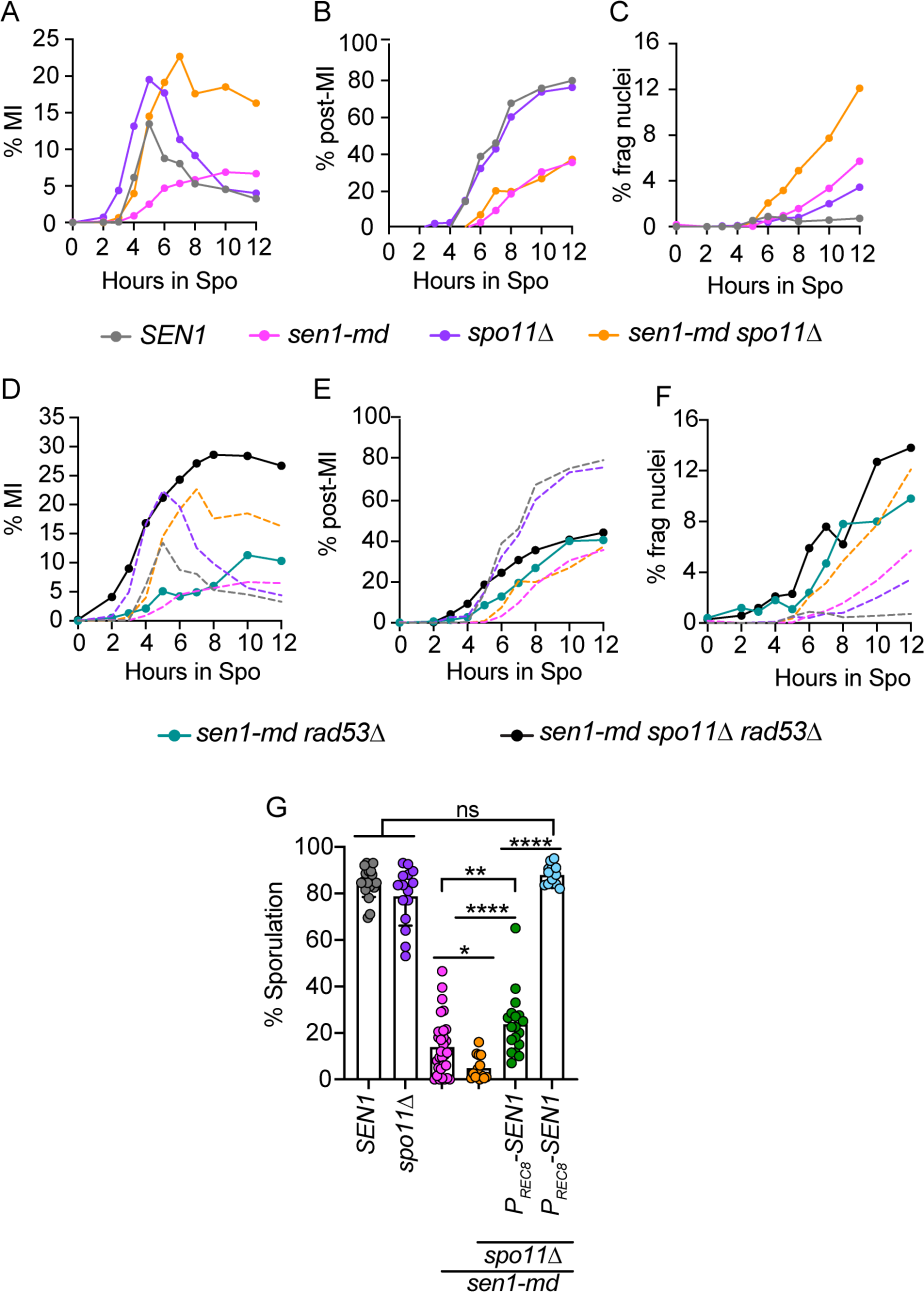
Meiotic progression and sporulation of *sen1-md* diploids containing *spo11∆.* *SEN1*, *sen1-md*, *spo11∆* (NH1055, *n* = 10) and *spo11∆ sen1-md* (NH2689, *n* = 15) diploids were induced to undergo meiosis and the average frequency of the indicated cell types was determined at various time points as described in Fig 3. The *SEN1* and *sen1-md* data for panels A-C are the same as Fig 3. **(A)** Cells that have completed MI; **(B)** Post-MI includes both MII cells and cells with fragmented nuclei (>4 DAPI foci). **(C)** Cells with fragmented nuclei. **(D-F)** Comparison of %MI, % post-MI and % cells with fragmented nuclei from *sen1-md rad53∆ sml1∆* (NH2843) and *sen1-md spo11∆ rad53∆ sml1∆* (NH2844) diploids with the timecourses shown in A-C. Dotted lines are the same as in A-C. **(G)** Different colonies from the diploids used in Panels A-C, *spo11∆ P*_*REC8*_*-SEN1* (NH2689::pNH410) and *spo11∆ P*_*SEN1*_*-SEN1* (NH2689::pBG28) were sporulated at 30°C on solid medium. Each dot represents a biological replicate for which the frequency of sporulated cells was determined for 200 cells. The data for *SEN1* and *sen1-md* from Fig 4 are repeated in all of the graphs. Error bars indicate the means and standard deviations. Statistical significance of differences between strains was determined using the Mann-Whitney test (* = *p* < 0.02; ** = *p* < 0.002; *** = *p* < 0.001; **** = *p* < 0.0001).

If Spo11-generated meiotic DSBs are responsible for the MRC-mediated prophase I delay in the *sen1-md* diploid, then deleting *SPO11* should rescue this delay. In fact, the *sen1-md spo11∆* diploid progressed faster through MI than *sen1-md*, consistent with the MRC no longer being triggered (Fig 7A), However, as no Spo11 DSBs have been made, the chromosomes should be intact and therefore enter MII as efficiently as *spo11∆*, but this was not the case (Fig 7B). Instead, the *sen1-md spo11∆* diploid exhibited a similar number of post-MI cells as *sen1-md*, but the number of fragmented nuclei was much higher and sporulation was significantly reduced (Fig 7B, 7C and 7G). The fragmented nuclei show that DSBs were present in the *sen1-md spo11∆* cells that were not directly created by Spo11.

Meiotic DSBs are a critical part of prophase I and so do not trigger the Rad9-Rad53-mediated DNA damage checkpoint, and their repair is instead regulated by Mek1 [66,92,119]. However, DSBs that persist past MI trigger the DNA damage checkpoint and delay entry into MII [120]. Therefore, one explanation for why *sen1-md spo11∆* cells did not efficiently proceed through MII would be if DSBs were present post-MI that triggered the *RAD53*-dependent DNA damage checkpoint. This idea was tested by looking at meiotic progression in *sen1-md* and *sen1-md spo11∆* diploids containing deletions of both *RAD53* and *SML1*, since *rad53∆* cells are inviable when *SML1* is present [121]. (For simplicity, the *rad53∆ sml1∆* genotype is referred as *rad53∆*).

This analysis revealed that *sen1-md* not only triggers the MRC during prophase I, it also triggers the DNA damage checkpoint post-MI. While the slow progression through MI was similar in the *sen1-md* and *rad53∆ sen1-md* diploids due to the MRC, the *rad53∆ sen1-md* cells entered MII more quickly than *sen1-md* (Fig 7D and 7E). Furthermore, there was an increase in the frequency of cells with fragmented nuclei in *rad53∆ sen1-md*, indicating that the absence of the post-MI DNA damage checkpoint allowed some DSBs to escape repair (Fig 7F).

An unexpected result was observed in the *rad53∆ sen1-md spo11∆* diploid. Since the DNA damage checkpoint does not function during prophase I, deletion of *rad53∆* should have no effect on the kinetics of progression through MI (Fig 7D) [120]. However, entry into MI was significantly faster and more efficient in *rad53∆ sen1-md spo11∆* compared to *sen1-md spo11∆* (Fig 7D). Significantly more *rad53∆ sen1-md spo11∆* cells progressed through MII than the *sen1-md spo11∆* strain, perhaps because more cells completed MI (Fig 7E). The frequency of cells with fragmented nuclei increased as well (Fig 7F). These observations suggest that in *sen1-md spo11∆* diploids, *RAD53* functions prior to prophase I in response to *SPO11*-independent DSBs to delay progression into prophase I and the first meiotic division.

In contrast to *NDT80-mid sen1-md* and *mek1∆ sen1-md*, *P*_*REC8*_*-SEN1* only weakly complemented the sporulation defect of *sen1-md spo11∆* (Fig 7G). However, the *sen1-md spo11∆* sporulation defect was completely rescued when *SEN1* was constitutively expressed using *P*_*SEN1*_*-SEN1*. This difference indicates that the timing of *SEN1* transcription matters in the absence of *SPO11* and argues that DSBs present in *sen1-md spo11∆* are temporally separable and occur earlier than the Spo11-generated DSBs in *sen1-md*.

### *SEN1* prevents accumulation of DNA:RNA hybrids during premeiotic S phase and prophase I

**Fig 8:**
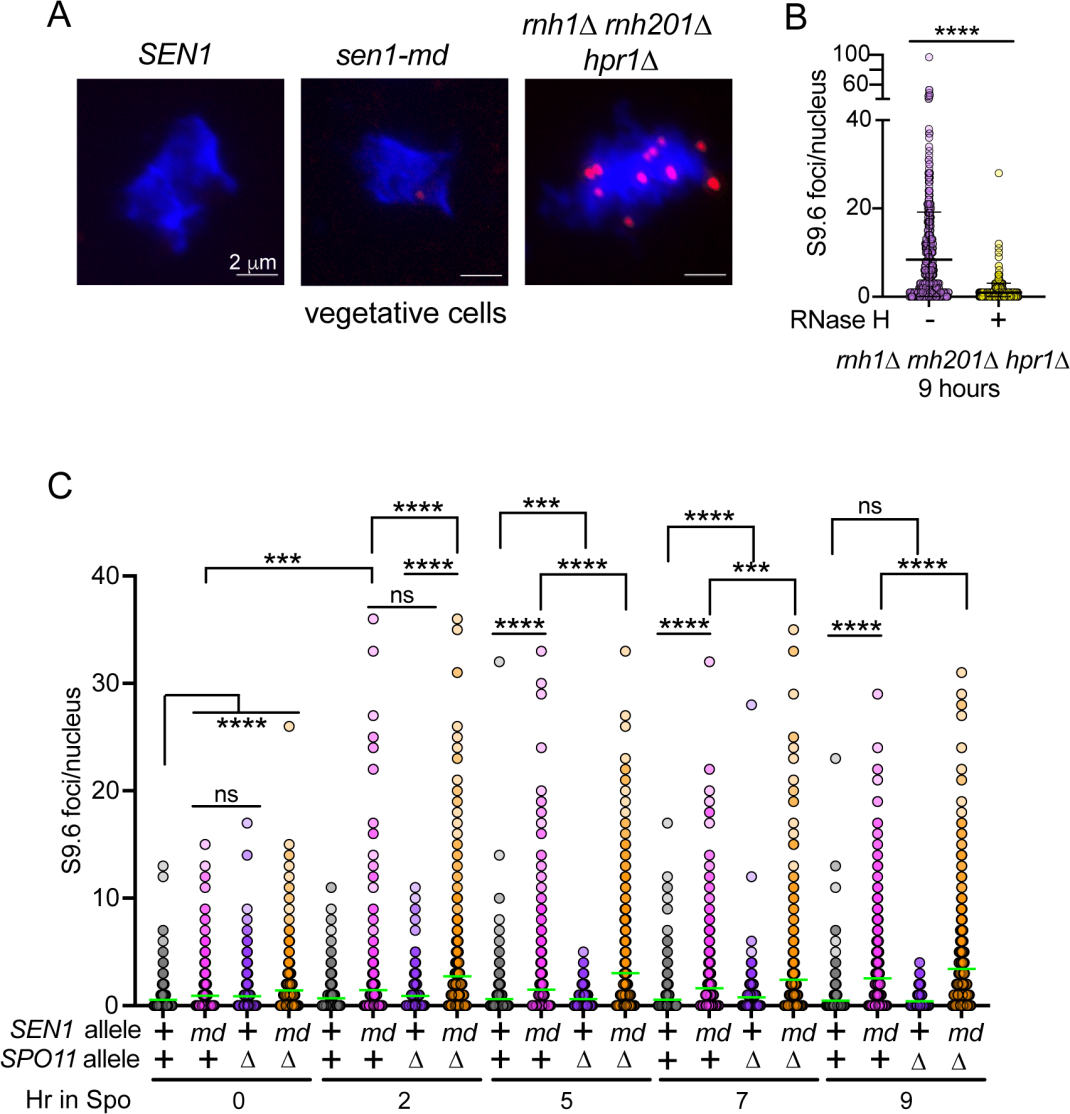
*SEN1* decreases DNA:RNA hybrids during meiosis. **(A)** Chromosome spreads were prepared from vegetatively growing *SEN1*, *sen1-md* and *rnh1∆ rnh201∆ hpr1∆* (LZY2919) diploids. The DNA was stained with DAPI (blue) and DNA:RNA hybrids were detected by immunofluorescence (red foci) using the S9.6 antibody. **(B)** Chromosome spreads from *rnh1∆ rnh201∆ hpr1∆* cells at the 9 hr meiotic timepoint were treated with either bovine serum albumin (-RNase H) or RNase H (+RNase H) and then stained with the S9.6 antibody (*n* = 2). **(C)** DNA:RNA hybrid foci were detected on chromosome spreads from *SEN1* (*n* = 4 or 5), *sen1-md* (*n* = 4, 5 or 6), *spo11∆* (*n* = 3) and *sen1-md spo11∆* (*n* = 3) at the indicated timepoints using the S9.6 antibody. *n* varies for some strains depending upon the timepoint. Two hundred spreads were examined for each biological replicate. Green bars indicate the mean values. Statistical significance was determined using the Mann-Whitney test (* = *p* < 0.02; ** = *p* < 0.002; *** = *p* < 0.001; **** = *p* < 0.0001).

Previous work demonstrated that in the absence of RNaseH activity, DNA:RNA hybrids form at the ends of Spo11-generated DSBs in prophase I, resulting in a meiotic progression delay and reduced spore viability [17,31]. To test whether *SEN1* similarly prevents DNA:RNA hybrid accumulation during meiosis, the frequency of DNA:RNA hybrids (some of which may be R-loops) was compared between *SEN1* and *sen1-md* at different times after transfer to Spo medium. DNA:RNA hybrids were detected as foci on chromosome spreads using the S9.6 antibody (Fig 8A) [32]. The *rnh1∆ rnh201∆ hpr1∆* diploid was used as a positive control as it has previously been shown that vegetative cells with this genotype exhibit numerous DNA:RNA hybrid foci using this antibody (Fig 8A) [17]. To further validate the assay, chromosome spreads from *rnh1∆ rnh201∆ hpr1∆* cells collected 9 hours after transfer to Spo medium were treated with RNase H prior to antibody staining. This treatment led to a significant reduction of S9.6 foci, confirming that the foci are DNA:RNA hybrids (Fig 8B).

The *sen1-md* diploid exhibited a statistically significant increase in S9.6 foci at the 0 hour timepoint compared to *SEN1* (Fig 8C). This observation suggests that the *sen1-md* mutant is hypomorphic in vegetative cells; that is, while viable, the reduced amount of Sen1 protein has an effect on R-loop levels. An even higher frequency of DNA:RNA hybrids was observed in *sen1-md* starting at 2 hours and remained high even after 9 hours in Spo medium (Fig 8C). At 5 and 7 hours, the *SEN1* diploid exhibited a significantly higher number of S9.6 foci compared to *spo11∆*, which is unable to make prophase I DSBs (Fig 8C). However, this difference was not present after 9 hours, as expected if the DSBs in the *SEN1* strain were repaired by then (Figs 5D and 8C). These results support the idea that the MRC is triggered in the *sen1-md* mutant by DNA:RNA hybrids present at the ends of *SPO11*-dependent DSBs.

A previous study used the S9.6 antibody to do ssDRIP-seq (single strand DNA:RNA hybrid immunoprecipitation) to map the specific locations of DNA:RNA hybrids in meiotic yeast cells in an *rnh1∆ rnh201∆* diploid [31]. At later timepoints (e.g., 5 hour), DNA:RNA hybrids were observed that corresponded to the positions of Spo11 oligos indicating meiotic DSBs. In addition, at an early timepoint (2 hour), DNA:RNA hybrids (proposed to be R-loops) were found at places where the transcription of highly transcribed genes collides head on with replication (TRCs) [31]. The frequency of these hybrids was increased in the *spo11∆* diploid.

To see if *SEN1* regulates the presence of R-loops during premeiotic S phase, S9.6 foci were examined after 2 hours in Spo medium. The timing of DNA replication in the *SEN1*, *sen1-md*, *spo11∆* and *sen1-md spo11∆* diploids was determined using flow cytometry. Replicating cells were present at the 2 hour timepoint in *SEN1*, but replication was delayed in the *sen1-md* mutant by an hour due to the delay in *IME1* transcription (S6 Fig). Consistent with the literature, the *spo11∆* diploid proceeded faster through premeiotic S phase, and this partially counteracted the *sen1-md* delay such that there were replicating cells after 2 hours in the *sen1-md spo11∆* strain (S6 Fig) [122]. At the 2 hour timepoint, the frequency of S9.6 foci was significantly increased in the *sen1-md spo11∆* strain compared to either the *sen1-md* or *spo11∆* mutant alone (Fig 8C). *SEN1* therefore functions in conjunction with *SPO11* to suppress the presence of DNA:RNA hybrids during premeiotic S phase.

## Discussion

### *SEN1* is a regulator of meiotic early gene expression

The *sen1-md* delay in meiotic progression was only partially rescued by *P*_*REC8*_*-SEN1*, revealing the unexpected result that *SEN1* regulates entry into meiosis. RNA-seq analysis showed that transcription of the *IME1* meiotic early gene master regulator was delayed in the *sen1-md* diploid, resulting in a coordinate delay in early and middle gene expression. Furthermore, *SEN1* directly affects transcription of *IME1* through regulation of the *IME1* promoter, because substituting the *IME1* promoter with the *CUP1* promoter allowed *sen1-md::P*_*REC8*_*-SEN1* to progress through meiosis with the same kinetics as *SEN1*. *IME1* transcription is negatively regulated by the presence of the non-coding RNA *IRT1*, which is downregulated in meiosis by the absence of Rme1 and the expression of the upstream non-coding RNA *IRT2* [80,82]. Given Sen1’s role in terminating non-coding RNAs, one hypothesis to explain these results would be if there was readthrough of the *IRT2* transcript into the *IRT1* region when *SEN1* function was impaired. To test this idea, individual transcripts starting in *IRT2* and continuing into *IRT1* would need to be detected. Towards this end, a protocol was developed for looking at non-coding RNAs present after 2 hours in Spo medium by dRNA sequencing.

The dRNA sequencing analysis confirmed that *IME1* transcripts were reduced in *sen1-md*, explaining why it takes longer for sufficient Ime1 to accumulate for cells to enter meiosis. While *IRT2* readthrough was not observed, ruling out our initial hypothesis, dRNA-seq revealed instead a dramatic change in the expression pattern of the *IME1* region. Novel antisense transcripts starting in *IRT1* and proceeding through *IRT2* were detected, as well as antisense transcripts that spanned a ribosomal protein gene upstream of *IRT2*. These results suggest that *SEN1* suppresses antisense transcription in this region, perhaps by premature transcriptional termination of the antisense RNAs. Further study is necessary to determine the mechanism by which *SEN1* suppresses antisense transcription upstream of *IME1*, as well as whether this aberrant transcription negatively affects *IME1* expression.

### *SEN1* functions prior to prophase I to prevent *SPO11*-independent DSBs

During S phase in vegetatively growing cells, R-loops accumulate at places where replication collides head-on with transcription (TRCs) [6,9,123–127]. When a TRC occurs, positive supercoils accumulate between the replication fork and transcription machinery, stalling both processes. The transcribed RNAs may then base pair with the template strand of the DNA to make R-loops [126]. Sen1 associates with the replisome and helps prevent R-loops from forming at TRCs both by displacing RNA Pol II as well as coordinating the activities of topoisomerases I and II to relieve topological stress [123,124,126,127].

R-loops also accumulate at TRCs during premeiotic S phase [31]. When the precise genomic locations of R-loops were mapped in an *rnh1∆ rnh201∆* SK1 diploid after transfer to Spo medium, an increase in R-loops at TRCs was observed at the 2 hour timepoint when premeiotic S was occurring [31,122]. No increase in R-loop signal was detected when the orientation of a specific TRC was changed such that replication and transcription were co-directional. The R-loops at premeiotic TRCs are distinct from the DNA:RNA hybrids formed at Spo11-generated DSBs in that they do not co-localize with Spo11 hotspots and their levels are reduced at later timepoints when chromosomes have fully synapsed [31]. Interestingly deletion of *SPO11* increased the frequency of R-loops at the premeiotic TRCs and these R-loops exhibited a similar distribution as the R-loops observed in the *rnh1∆ rnh201∆* diploid [31].

Several observations support the idea that *SEN1* also plays a role in preventing/removing R-loops at TRCs during premeiotic S phase. (1) *sen1-md* diploids exhibited a severe sporulation defect, in part because some cells arrest in prophase I due to unrepaired DSBs triggering the MRC. If the *sen1-md* sporulation defect was due solely to unrepaired Spo11 DSBs, then *spo11∆* should have rescued sporulation in the *sen1-md* diploid, but this was not the case. (2) In the *sen1-md spo11∆* strain, the MRC was abrogated as expected, with an increase in MI cells compared to *sen1-md*. Surprisingly, most of the cells that progressed past MII exhibited fragmented nuclei, demonstrating the presence of DSBs. These DSBs were not generated directly by Spo11 since the protein is not there. (3) Progression through MI was faster and more efficient when the DNA damage checkpoint kinase *RAD53* was deleted from *sen1-md spo11∆*, suggesting that DSBs formed during premeiotic S phase are triggering this checkpoint prior to prophase I. (4) *P*_*REC8*_*-SEN1* only weakly rescued the sporulation defect of *sen1-md spo11∆*, while *P*_*SEN1*_*-SEN1* rescued completely, arguing that the timing of *P*_*REC8*_*-SEN1* expression was too late in the *sen1-md spo11∆* diploid to effectively complement *sen1-md*. In contrast, *P*_*REC8*_*-SEN1* expression was not too late to allow repair of Spo11-generated DSBs in *NDT80-mid sen1-md* or *mek1∆ sen1-md* strains. These results suggest that *SPO11*-independent DSBs arise at an earlier time than *SPO11*-dependent prophase I DSBs. (5) After 2 hours in Spo medium, DNA:RNA hybrid foci were increased in *sen1-md spo11∆* compared to either *sen1-md* or *spo11∆*. The latter finding is informative because *spo11∆* diploids do not make the prophase I DSBs that are used for recombination and therefore the S9.6 foci in the *sen1-md spo11∆* cannot be attributed to DNA:RNA hybrids at resected Spo11-dependent DSB ends.

We propose that the *SPO11*-independent DSBs in the *sen1-md spo11∆* diploid arise because (1) R-loops formed at TRCs during premeiotic S phase get converted to DSBs that trigger the DNA damage checkpoint and (2) *SEN1* is not present to prevent/eliminate R-loops at premeiotic TRCs. Sen1 could help prevent R-loop formation by removing RNA Pol II from TRCs or by coordinating topoisomerase I and II activity as it does during vegetative S phase. However, *SEN1* is not sufficient to completely prevent R-loop formation at TRCs, otherwise the R-loop frequency at TRCs would not increase in *rnh1∆ rnh201∆* diploids where *SEN1* is functional [31]. Therefore, it is also possible that Sen1 may disassemble R-loops at premeiotic TRCs using its helicase activity. The multi-functional roles that *SEN1* may play at premeiotic TRCs could explain why *sen1-md* is more defective in sporulation and spore viability than the *rnh1∆ rnh201∆* mutant, since RNase H1/2 can only remove R-loops but not prevent their formation [17,31].

An interesting question is what *SPO11* is doing at TRCs in premeiotic S phase. During wild-type meiosis, Spo11 is recruited to chromosome axes after DNA replication where it generates prophase I DSBs [128–130]. During premeiotic S phase, Spo11 may generate DSBs to relieve the topological constraints of the positive supercoils on unreplicated DNA, which would be a novel function for this highly conserved endonuclease.

The fact that progression through MI in *NDT80-mid sen1-md*, *mek1∆ sen1-md* and *sen1-md spo11∆* diploids was similar indicates that *SPO11*-independent DSBs do not trigger the MRC. Spo11-generated DSBs occur on the chromosome axes, after which the Mec1/Tel1 checkpoint kinases phosphorylate Hop1 [48]. Mek1 binds to phosphorylated Hop1 via its FHA domain and then activates itself by phosphorylation of the Mek1 activation loop in *trans* [50,51,131]. We propose that the *SPO11*-independent DSBs arise from processing of persistent R-loops formed at TRCs during premeiotic S phase and thus are likely not forming on chromosome axes, in which case Mek1 (and therefore the MRC) would not be activated [50,51,131]. A similar phenomenon was observed in *C. elegans* mutants lacking RNase H activity where R-loops accumulated in the germ line [26]. An increased frequency of DSBs was observed in *rnh-1.0 rnh-2; spo11* worms compared to *spo11* alone and those DSBs failed to trigger the DNA damage checkpoint [26]. *SPO11*-independent DSBs have also been observed during premeiotic S phase in mouse spermatocytes [132]. Therefore, it is possible that a non-canonical function for Spo11 in regulating R-loops during meiosis is conserved.

### *SEN1* promotes the repair of *SPO11* DSBs during prophase I

Several studies have shown that DNA:RNA hybrids can form on the resected ends of DSBs in a variety of species both in vegetative and meiotic prophase I cells [13,14,16,17,26,31,33,34]. In budding yeast meiosis, DNA:RNA hybrids that accumulated at the resected ends of DSBs in an *rnh1∆ rnh201∆ hpr1∆* diploid impaired recombinase binding and interfered with DNA repair [17].

Our work indicates that *SEN1* also functions to promote repair of *SPO11* DSBs. Meiotic progression in *sen1-md* was delayed due to the MRC that is triggered by Spo11-generated DSBs [50–53]. Furthermore, when *sen1-md* diploids were allowed to progress through meiosis without the MRC-mediated delay, nuclei were fragmented, indicating the presence of broken chromosomes. DNA:RNA hybrid foci were elevated in *sen1-md* strains throughout prophase I. Chromosome synapsis was defective in the *sen1-md* mutant but this defect was significantly improved when *SEN1* was expressed using the *REC8* promoter. We propose that *SEN1* removes DNA:RNA hybrids from the ends of Spo11 prophase I DSBs and that this removal is necessary for recombinases to bind, similar to what has been observed in *rnh1∆ rnh201∆ hpr1∆* diploids [17], In this case, the synapsis defect is an indirect effect of a problem with DSB repair.

Interestingly, despite the strong synapsis defect, COs were reduced only by ~25% in the *sen1-md* diploid. This value is not very different from the 40% reduction in COs in the *rnh1∆ rnh201∆ hpr1∆* diploid (Fig 5) [17]. One explanation for the relatively minor CO defects is that RNase H1/2 and Sen1 function redundantly in removing the DNA:RNA hybrids at the ends of DSBs, although the *rnh1∆ rnh201∆ sen1-md* diploid was too sick to directly test this idea. One difference between the RNaseH mutant is that it decreased both COs and NCOs, whereas only COs were decreased by *sen1-md* (Fig 5) [17]. The ZMM crossover pathway is required for chromosome synapsis [64,102,106]. An intriguing idea is that Sen1 specifically removes DNA:RNA hybrids from DSB ends that are destined to be repaired by the ZMM pathway. In this case, most of the COs observed in the *sen1-md* mutant would be generated by the action of structure-selective nucleases such as Mus81-Mms4 that do not contribute to synapsis. RNase H may instead remove DNA:RNA hybrids from DSBs that are processed by other pathways to form either COs or NCOs. Further work is necessary to test this idea.

## Methods

### Strains and media

All the strains used in this study were derived from the SK1 background. Genotypes of each strain are listed in S1 Table. Growth media are described in [133]. Sporulation (Spo) medium consisted of 2% potassium acetate. All experiments were carried out at 30°C.

Gene deletions were made using polymerase chain reaction (PCR) generated fragments to replace open reading frames (ORFs) with either *kanMX6* (confers G418 resistance), *natMX4* [confers nourseothricin (NAT) resistance], or *hphMX4* (confers Hygromycin B resistance) using the plasmids pFA6a-kanMX6, p4339, or pAG32, respectively. Deletions were verified by PCR using a forward primer upstream of the deleted ORF and a reverse primer within the drug marker. In addition, the absence of the wild-type gene was confirmed by failure to detect a PCR product using the same forward primer and a reverse primer in the ORF. For the *ndt80∆::hphMX4* haploid parents of AM6697, flanking primers generated different sized PCR products for the *NDT80* and *ndt80∆::hphMX4* alleles.

To make *sen1-md* strains, a 2.5 kb fragment was amplified from pMJ787 containing *kanMX6* 133 bp upstream of *P*_*CLB2*_*-3xHA*. This fragment was designed with homologies such that it replaces the 50 bp immediately upstream of the *SEN1* ATG with the *CLB2* promoter. G418^R^ haploid transformants of both mating types were screened by colony PCR to confirm the insertion of the fragment directly upstream of *SEN1* using a forward primer located 814 bp upstream of *SEN1* and a reverse primer 740 bp downstream of the *SEN1* start codon.

The *P*_*REC8*_*-SEN1* and *P*_*REC8*_*-sen1-∆N URA3* integrating plasmids, pNH410 and pBG27, respectively, were digested with PshAI to target integration to codon 1811 of *SEN1* and transformed into the *sen1-md* diploid, NH2667. Integration of the plasmids was confirmed by complementation of the *sen1-md* sporulation defect and/or immunoblots probed with α-Sen1 antibodies.

The *sen1-md NDT80-mid* diploid, NH2667::pNH317^2^ was created by transforming the haploid parents of NH2667 with the *URA3 NDT80-mid* integrating plasmid, pNH317, digested with SnaBI to target integration upstream of the *NDT80* ORF (note that *NDT80-mid* is dominant to *NDT80*) [52]. Proper plasmid integration was confirmed by PCR with a forward M13 primer present in the vector sequence with a reverse primer 1.9 kb upstream of the *NDT80-mid* ORF.

Diploids containing *rad53∆::kanMX6* were constructed in several steps. For *rad53∆* strains to be viable, the *SML1* gene must be deleted [121]. The *SML1* gene was replaced with 3HA epitopes using pMPY-3HA as a template to amplify a *3HA-URA3-3HA* cassette flanked by sequences directly upstream and downstream of the *SML1* ORF. This cassette was transformed into S2683 selecting for Ura^+^ colonies. The cassette was popped out using 5-fluororotic acid, which selects against cells containing the *URA3* gene, replacing the *SML1* ORF with the *3HA* sequence [134,135]. *RAD53* was then deleted using *kanMX6*. This haploid was crossed to NHY1210 *sen1-md spo11∆::natMX4* (a haploid parent of NH2689). Spore colonies from tetrad dissection of this diploid were screened for G418 resistant non-parental ditypes that should contain both *rad53∆::kanMX6* and *kanMX6::P*_*CLB2*_*-SEN1*. The presence of both genes was confirmed by PCR. For *sml1∆::3HA*, the PCRs used primers that flank the *SML1* ORF. The NH2840-23-3 and NH2840-71-1 segregants were NAT sensitive and therefore contained *SPO11*. The NH2840-23-4 and NH2840-33-4 segregants were resistant to NAT and therefore contained *spo11∆::natMX4*. The appropriate haploid pairs were mated to make NH2843 and NH2844, respectively.

To make a *P*_*CUP1*_*-IME1 sen1-md* diploid, a PCR fragment containing *kanMX:P*_*CLB2*_*-SEN1*^*741*^ was amplified using genomic DNA from NH2667 as the template and primers located 571 bp upstream of *kanMX* and 741 bp downstream of the *SEN1* ATG. This fragment was transformed into the *P*_*CUP1*_*-IME1* haploids, 39806 and 39823, and the presence of the *sen1-md* allele confirmed by PCR. The haploids were then mated to generate the NH2842 diploid. The *P*_*REC8*_*-SEN1* allele was integrated into NH2842 using PshAI digested pBG45.

The *sae2∆ sen1-md* diploid, NH2846, was constructed by first replacing *SAE2* with *natMX4* in the haploid parents of NH716. The resulting two *sae2∆::natMX4* haploids were transformed with the *kanMX::P*_*CLB2*_*-SEN1*^*741*^ fragment described above to introduce the *sen1-md* mutation. The two haploids were then mated to make NH2846.

### Plasmids

The genotypes of plasmids are listed in S2 Table. Any sequences amplified by PCR were sequenced in the final plasmids by the Stony Brook University DNA Sequencing Facility or by Plasmidsaurus (https://www.plasmidsaurus.com/).

The *SEN1* gene was fused to the *REC8* promoter in pNH257 to make pNH410 using the NEBuilder HiFi DNA Assembly Master Mix (hereafter referred to as Gibson assembly or GA) (New England Biolabs E2621). A 2μ *SEN1* plasmid containing a genomic fragment from chromosome XII with the coordinates 989631*-*1000863 was used as the template for PCR [136]. The 6696 bp *SEN1* ORF was amplified as three separate fragments of approximately 2 kb. The SEN1a fragment contains 25 bp of homology to the BamHI cut site of pNH257 (immediately downstream of *P*_*REC8*_) fused to *SEN1* codons 1–790. SEN1b contains codons 783–1555 and SEN1c contains codons 1547–2231 along with 210 bp of 3’ untranslated sequence and 25 bp of homology to the EcoRI cut site of pNH257. The three PCR fragments were incubated with BamHI/EcoRI-digested pNH257 in a GA reaction following the manufacturer’s instructions. The *P*_*REC8*_*-SEN1* allele was moved into a *TRP1* integrating vector by subcloning a 7.2 kb XhoI/NotI fragment from pNH410 into XhoI/NotI digested pRS304 to make pBG45. The *P*_*REC8*_*-sen1-∆N* allele in pBG27 lacks the first 1003 codons of *SEN1*. pNH410 was used as the template to generate a 1691 bp PCR fragment containing homology to the EcoRI site on pNH257 along with an ATG and *SEN1* codons 1004–1555. This fragment was then joined to the SEN1c fragment and ligated into pNH257 using GA as described for pNH410. The *P*_*SEN1*_*-SEN1* plasmid, pBG28, was constructed using a SEN1a fragment that had 25 bp of homology to the NotI site in pRS306 fused to a 2.8 kb sequence containing 0.8 kb located immediately upstream of the *SEN1* ORF, as well as the first 791 codons of the gene. The SEN1b fragment was the same as for pNH410, while the SEN1c fragment contained the same fragment of *SEN1* with 192 bp 3’ untranslated sequence and 3’ homology to the SalI site in pRS306.

### Timecourses

Liquid sporulation was performed as described in [133] with the following changes. Diploids were streaked out from the -80°C freezer onto YPD medium supplemented with complete powder [133] and incubated at 30^o^C for 2–3 days. Single colonies were inoculated into 8 mL YPD and incubated at 30^o^C for 24 h. 1.0 mL and 1.7 mL of inoculum were then diluted into 200 mL YPA in two 2 L flasks and incubated at 240 revolutions per minute (RPM) for 18 hours at 30^o^C. The optical density at 660 nm (OD_660_) of a 2-fold dilution in YPA of the culture was read using a SPECTRONIC 200 spectrophotometer. Diluted cultures with OD_660_ readings between 1.6 and 2.3 were pelleted, washed with water and resuspended in the volume of Spo medium required for a cell density of 3 x 10^7^ cells/mL in 2 L flasks. The cells were incubated in a 30°C shaker rotating at 250 RPM. At each timepoint, cells were fixed with a 1/10 volume of 37% formaldehyde for DAPI staining (Vector Laboratories H-1200-10). Formaldehyde-fixed cells were processed for whole cell immunofluorescence as described in (Ziesel *et al.* 2022). For protein and RNA sequencing samples, 5 ml sporulating culture were pelleted in a 15 ml conical tube, the supernatant was discarded, and the cell pellets stored at -80°C. For DNA physical analysis, 10 mL culture was added to 1 mL 0.5 M EDTA and 10 mL 100% ethanol and stored at -20°C. For nuclear spreads, 6.7 mL of sporulating culture were immediately processed as described in [137]. Cells were prepared for flow cytometry analysis as described in [138]. The cells were processed by the Stony Brook University Flow Cytometry Facility using a Becton Dickenson LSRFortessa flow cytometer. The data were analyzed using Beckman Coulter Kaluza software.

To induce *IME1* transcription in the *P*_*CUP1*_*-IME1* diploids, cells were transferred to Spo medium for 2 hours after which 100 mM CuSO_4_ (Sigma #209198) was added to a final concentration of 50 μM.

### Immunoblots and antibodies

Total protein was isolated from frozen cell pellets and utilized for immunoblot analyses as described in (Weng, Wan et al. 2024). The sources and conditions used for various antibodies are listed in S3 Table. The Sen1 antibody was generated by Covance Research Products (now Labcorp Drug Development) in a guinea pig using the peptide Ac-CFSDDVSFIPRNDEPEIK-amide (amino acids 2002–2019).

### Nuclear spreads

**For localization of meiotic axis and central region proteins of the SC (**Fig 6**):** Strains homozygous for *ndt80::∆hphMX4* were cultured overnight in BYTA (1% yeast extract, 2% bactopeptone, 1% potassium acetate, 50 mM potassium phthalate) then sporulated in 1% potassium acetate for 5 hours. Nuclei from these cells were surface-spread on glass slides and imaged as described in [139]. Affinity purified rabbit anti-Zip1 (1:150), mouse anti-Gmc2 (1:800), and rabbit anti-Red1 (1:200) were used [59]. Secondary antibodies conjugated with Alexa Fluor dyes (Jackson ImmunoResearch) were used at a 1:200 dilution. Microscopy and image processing were performed using a Deltavision RT imaging system (General Electric) adapted to an Olympus (IX71) microscope.

**For localization of DNA:RNA hybrids (Fig 8):** Meiotic cultures were spheroplasted and their nuclei spread and immunostained as described in [137]. Slides treated with 5 units of RNase H (New England Bioscience M0297L) were incubated at 37^o^C for 1 h in a moist slide box after blocking and then washed with 1xTBS prior to incubation with antibodies. Spreads were incubated with antibodies at 4^o^C in a moist slide box. Slides were first stained for DNA:RNA hybrids with the mouse DNA:RNA hybrid antibody, S9.6, (Kerafast ENH001) diluted 1:1000 in 1% BSA and incubated overnight. After washing slides as described, goat α-mouse IgG-Alexa 594 (Fisher A32742) secondary antibody diluted 1:1000 in 1% BSA was applied to slides and incubated for two hours. Micrographs were taken with a ZEISS Axio Imager.Z2 with a Zeiss Plan-Apochromat 100X objective and analyzed using ZEN 3.5 software.

### Physical analysis of DSBs and repair products

Genomic DNA was isolated from ethanol-fixed sporulation samples using the MasterPure Yeast DNA Purifiation Kit (Biosearch Technologies MPY80200) and quantified with the Qubit 1x dsDNA HS Assay Kit (Invitrogen Q33231). Digestion of samples, gel electrophoresis, Southern blotting, and hybridization with radiolabeled probe for analysis of the *HIS4LEU2* hotspot were performed as described in [140] with the following changes: the blotted membrane was incubated in prehybridization solution [0.5% SDS, 0.5 mg/mL sheared salmon sperm DNA, 6xSSC (29.22 mg/mL sodium chloride, 14.7 mg/mL sodium citrate, HCl to pH 7.0)] at 68^o^C for 2 hours. ^32^P-labeled probe with at least 1x10^6^ counts per minute was added to the hybridization solution (0.5% SDS, 0.5 mg/mL sheared salmon sperm DNA, 6xSSC, 0.1 mg/mL dextran sulfate) and incubated with the membrane for 16 h at 68^o^C. The membrane was washed at 68^o^C three times for 30 minutes with ~40 mL of Wash Buffer 1 (2x SSC, 0.1% SDS) and then twice with Wash Buffer 2 (0.2x SSC, 0.1% SDS). It was then washed at room temperature for 5 minutes each with Wash Buffer 2 and Wash Buffer 3 (0.1x SSC). The blots were imaged using a FujiFilm FLA-7000 phosphoimager and Multi-Gage software.

### Illumina RNA sequencing analysis

After growth in YPA at 30°C, the *SEN1* and *sen1-md* diploids were transferred to Spo medium at a density of 3 X 10^7^ cells/ml and 5 ml aliquots were taken at various timepoints, pelleted and frozen at -80°C. The cell pellets were sent to GENEWIZ (now Azenta) for RNA extraction and polyA RNA enrichment. The kit used to prepare the libraries was the NEBNext Ultra RNA Library Prep, PolyA (New England Biolabs). The adapter sequences used to generate the RNA sequencing data were:

Read1: AGATCGGAAGAGCACACGTCTGAACTCCAGTCAC
Read2: AGATCGGAAGAGCGTCGTGTAGGGAAAGAGTGTA

The RNA sequencing data were mapped to the yeast genome using Bowtie 1.1.2 and custom Python scripts with Yeast PacBio 2016 as the reference genome (https://yjx1217.github.io/Yeast_PacBio_2016/data/) [141, 142]. Fragments/kb transcript/million (fpkm) values were determined using TopHat2 [143] which were then used to calculate the standard scores (Z-scores) for each gene using the formula (fpkm_n_ - fpkm_ave_)/SD, where n = timepoint, fpkm_ave _= average of the fpkm values for each timepoint and SD is the standard deviation. Genes were clustered based on their pattern of Z values using Clustal 3.0 and then visualized as a heat map plotted by JavaTree. The *sen1-md* strain was not weighted and these genes were “passengers” whose location was set by the *SEN1* cluster. The Illumina RNA sequencing data are available in the National Institutes of Health Gene Expression Omnibus (GEO) with the accession number GSE292703. (https://www.ncbi.nlm.nih.gov/geo/query/acc.cgi?acc=GSE292703).

### Nanopore dRNA sequencing analysis

#### *In vitro* transcription of biotinylated antisense rRNA probes.

*In vitro* transcription of biotinylated antisense rRNA probes was performed using modifications to the approach described in [144]. Briefly, primers were engineered to target the 25S, 18S, 5.8S, and 5S rDNA genes (*RDN25, RDN18*, *RDN58*, *RDN5*, respectively), with the reverse primers containing the T7 promoter. Because of the larger sizes of the 25S and 18S rDNA genes, primers were engineered to amplify 200 bp regions within the 5’ and 3’ ends of each gene. Primer sequences and the pairs used for each PCR fragment are listed in S4 Table. The rDNA genes were PCR amplified from *S. cerevisiae* genomic DNA (Novagen #69240) using KOD HotStart DNA Polymerase (Novagen #71086) and the PCR products were purified using standard Phenol:Chloroform:Isoamyl alcohol extraction followed by ethanol precipitation.

To generate the biotinylated antisense probes, 1 μg of amplified rDNA fragment was transcribed using the HiScribe® T7 High Yield RNA Synthesis Kit (New England Biolabs #E2040S;). Biotin was incorporated into nascent RNA transcripts using a 1:3 ratio of Biotin-16-UTP (Roche #11388908910):UTP. Transcription reactions (20 μL) were incubated at 37°C for 2 hours. To degrade the PCR fragments, 1 μL 20 U/μL DNase I (Worthington #LS006333) and 2.33 μL of 10x DNase I Buffer [500 mM Tris-HCl (pH 7.6), 10 mM MgCl_2_, 10 mM CaCl_2_] were added and the reactions incubated at 37°C for 20 minutes. Biotinylated antisense rRNAs were then purified by ethanol precipitation. The resulting pellets were resuspended in Molecular Grade water (Cat#: 46-000-CM; Corning). The RNA in each probe was quantified using the Qubit RNA HS Assay kit (Life Technologies #Q32852) and diluted to 2 μM using Molecular Grade water. For the 25S and 18S probes, both the 5’ and 3’ end probes were combined. To simultaneously deplete all four rRNAs, the 6 biotinylated RNAs (5’ and 3’ ends of *RDN25* and *RDN18*, *RDN58* and *RDN5*) were combined in Molecular Grade water, each at a 2 μM concentration.

#### Depletion of rRNA from total RNA extracts.

Two biological replicates of *SEN1* and *sen1-md* were incubated for two hours after transfer to Spo medium. Five mL cells (approximately 1.5x10^8^ cells) were harvested by centrifugation at 2851 x *g* for 5 minutes at 4°C. The cell pellets were washed with 1 mL Molecular Grade water to remove any residual medium. Cells were then centrifuged at 16,000 x *g* for 15 seconds at 4°C and the supernatants were removed. Pellets were flash frozen in liquid nitrogen and stored at -80°C. Total RNA was extracted using the RiboPure RNA Purification Yeast kit (Life Technologies #AM1926). Total RNA quality was checked by agarose gel electrophoresis and quantified using the A_260_ absorbance reading. For depletion of rRNA from total RNA, the approach described in [144] was modified. To hybridize the biotinylated antisense probes, 12.5 μg of total RNA were incubated with a mixture containing 20 pmol 25S antisense probe and 10 pmol each of 18S, 5.8S, and 5S antisense probes. Additionally, 20 μL of 10x Hybridization Buffer [500 mM Tris–HCl, pH 7.5 and 1 M NaCl] was added to each reaction and the reaction volume was brought up to 200 uL using Molecular Grade water. The hybridization reactions were incubated in a PCR machine (with the lid temperature at 85°C) using the following protocol: (1) 75°C for 2 min; (2) 70°C for 2 min; (3) 65°C for 2 min; (4) 60°C for 2 min; (5) 55°C for 2 min; (6) 37°C for 2 min; (7) 25°C for 2 min; (8) 4°C hold.

To deplete the hybridized rRNA using the biotinylated antisense probes, 800 μL of well mixed Dynabeads MyOne Streptavidin C1 (Invitrogen #65002) were added to each reaction and then pelleted using a DynaMag-2 magnet (Life Technologies #12321D). To prepare the magnetic beads, the storage buffer was first removed and the beads were washed three times with 500 μL 1x B&W buffer [5 mM Tris–HCl, 0.5 mM EDTA, 1 M NaCl], and resuspended in 365 uL of 2x B&W buffer. Hybridization reactions were added to the resuspended beads and incubated at room temperature for 15 minutes, followed by 50°C for 5 minutes. The beads were applied to a magnet for 5 minutes at room temperature to allow precipitation, and the supernatants containing the rRNA-depleted material were removed to new microfuge tubes. The remaining RNA was purified by adding 1.8x volume of RNAClean XP beads (Beckman Coulter #A63987) followed by incubation at room temperature for 5 minutes. The beads were pelleted with a magnet and washed three times using freshly made 70% ethanol before the purified RNA was eluted using 15 μL of Molecular Grade water. rRNA depletion efficiency was analyzed by gel electrophoresis using a 1.0% Agarose/ 1xTAE/ 1% Bleach gel.

#### Nanopore library preparation and sequencing.

To prepare samples for dRNA sequencing, the RNAs remaining in the rRNA-depleted sample were first polyadenylated using *E.coli* PolyA Polymerase (New England Biolabs #M0276S) by incubation for 1.5 minutes at 37°C. The reactions were quenched using 10 mM EDTA and purified by addition of 1.8x volume of RNAClean XP beads and incubation at room temperature for 5 minutes. The beads were pelleted with a magnet and washed two times using freshly made 70% ethanol and RNA was eluted using 10 μL of Molecular Grade water.

A library from the polyadenylated RNAs was prepared using the dRNA sequencing kit [Oxford Nanopore Technologies)(ONT) #SQK-RNA004] following the manufacturer’s instructions. Sequencing was performed using FLO-MIN004RA flow cells ONT #FLO-MIN004RA) on a MinION MK1B device (ONT #MIN-101B)) for 72 hours for each sample until 9–10 million reads were sequenced.

### Data processing

Base-calling was conducted on a custom Linux workstation equipped with an AMD Threadripper PRO 5955WS CPU, dual Nvidia RTX 4090 GPUs (MSI V510-007R), and 256 GB of RAM. The resulting FASTQ files were analyzed using the Epi2Me Labs wf-transcriptomes Nextflow pipeline (version 25.04.06) (https://nanoporetech.com/products/analyse/epi2me) with the -direct_rna option enabled. Reads were aligned to the SK1 reference genome using minimap2 with a preset override of -x splice -uf -k 14 -G1k, optimized for direct RNA reads with short introns. The reference genome was indexed in minimap2 with the -k 15 setting. The workflow produced aligned BAM files for downstream analysis.

The genomic coordinates of CUTs, SUTs, and ORF-T were originally annotated based on the S288C genome (version V56, 2007-04-06) [87]. These coordinates were first converted to genome version V64 (2011-02-3) using liftOver and subsequently remapped to the SK1 genome. This was achieved by retrieving the DNA sequences corresponding to the annotated regions with bedtools getfasta and aligned to the SK1 genome using minimap2. The resulting SAM alignment files were converted to sorted and indexed BAM files with samtools, which were then transformed to BED format using bedtools bamtobed for downstream analysis.

Differential gene expression was performed as follows. Read count quantification was determined using featureCounts (v2.1.1; Subread package). Reads were assigned to annotated ORF-T features defined in the SK1 genome using the following options: -F SAF-format annotation) and -O (minimum MAPQ > 20). The resulting count matrix was analyzed in R Studio (version 2025.09.1) with the DESeq2 package. Raw counts from two *SEN1* and two *sen1-md* dRNA-seq replicates were normalized for library size, and differential expression analysis was performed using DESeq2’s negative binomial model with Wald test statistics. Genes with a false discovery rate (FDR) < 0.01 and an absolute log_2_ fold change > 1 were considered signficantly differentially expressed.

To generate the normalized read count plots shown in Fig 3D and 3G, BAM alignments from replicates samples of *SEN1* and *sen1-md* were merged using samtools merge. Primary mapped reads were then extracted from the merged BAM files using samtools view with option -F 0x904 for regions chrXIV:173000-184300 and chrX:603000-609000, respectively. The filtered BAM files were converted to BED format and sorted first by the 3’-end position and then by 5’-end position of each read. Because the total number of aligned reads in the wild-type sample (15,252,836) were larger than that in *sen1-md* (12,637,870), the step size for individual reads was set at 1 for *SEN1* and 1.2 for *sen1-md* to normalize read density across samples. Datagraph was used to plot these figures.

To generate the heatmaps shown in Figs 3E, 3F, S3C, and S3D, deeptools bamCoverage was used to calculate normalized read coverage with the parameters --normalizeUsing CPM, --samFlagExclude 16 (for Watson strand) or –samFlagInclude 16 (for Crick strand), -bs 1, --effectiveGenomeSize 12053285. The resulting bigwig files were then used to generate the matrix files with the deeptools computeMatrix function, followed by heatmap visualization using deeptools plotHeatmap. The Nanopore dRNA sequencing data have been deposited in GEO with the accession number GSE310121. https://www.ncbi.nlm.nih.gov/geo/query/acc.cgi?acc=GSE310121.

Statistical analysis of the direct RNA-seq data was performed using DESeq2’s negative binomial model and Wald test statistics [DOI 10.1186/s13059-014-0550-8]. Genes with an adjusted *p*-value (i.e., false discovery rate) < 0.01 and an absolute log2 fold change > 1 were considered significantly different in our analysis. Under these criteria, *RAD50* is not significantly changed, as it shows a log2 fold change of 0.61 in *sen1-md* compared with *SEN1* and an adjusted *p*-value of 0.0191, which does not meet our predefined significance thresholds (Fig 3D). *IME1* transcript levels were determined to be significantly reduced in *sen1-md* relative to *SEN1* based on DESeq2’s negative binomial model and Wald test statistics. *IME1* shows a log2 fold change of –1.25 and an adjusted *p*-value of 5.56 × 10 ⁻ ⁸. Therefore, the decrease in *IME1* expression is statistically significant under our defined criteria (Fig 3G).

## Supporting information

S1 Data Contains the data and calculations used for all of the numerical data presented in Figures. (XLSX)

S2 Data Contains the data for the heatmaps in Fig 3E: *SEN1* CUT. (GZ)

S3 Data Contains the data for the heatmaps in Fig 3E: *sen1-md* CUT. (GZ)

S4 Data Contains the data for the heatmaps in Fig 3F: *SEN1* SUT. (GZ)

S5 Data Contains the data for the heatmaps in Fig 3F: *sen1-md* SUT. (GZ)

S6 Data Contains the data for the heatmaps in S3C Fig: *SEN1* ORF-T. (GZ)

S7 Data Contains the data for the heatmaps in S3C Fig: *sen1-md* ORF-T. (GZ)

S8 Data Contains the data for the heatmaps in S3D Fig: *SEN1* rORF-T. (GZ)

S9 Data Contains the data for the heatmaps in S3D Fig: *sen1-md* rORF-T. (GZ)

S1 Table *Saccharomyces cerevisiae* strains. (DOCX)

S2 Table Plasmids. (DOCX)

S3 Table Antibodies. (DOCX)

S4 Table Primers for amplifying ribosomal DNA sequences. (DOCX)

S1 Fig Immunoblots using a newly developed α-Sen1 antibody. (A) The *sen1-md* diploid, NH2667, was transformed with an integrating plasmid containing either *P*_*REC8*_*-SEN1* (pNH410) or *P*_*REC8*_*-sen1-∆N* (pBG27) and induced to undergo meiosis. Protein samples from the indicated timepoints were probed with α-Sen1 antibodies. α-Arp7 antibodies were used to detect Arp7 as a loading control. Numbers on the right indicate the positions of molecular weight markers in kiloDaltons. The black line indicates that the same samples were run on two different gels and probed with different antibodies. “Hr” refers to hours in Spo medium. (B) Timing of expression of various meiosis-specific proteins. Protein extracts from meiotic timecourses using *SEN1*, *sen1-md* and *P*_*REC8*_*-SEN1* strains were probed with antibodies against the indicated proteins. “pHop” and “pHed1” indicate phosphorylated Hop1 and Hed1, respectively.
(TIF)

S2 Fig Monitoring meiotic progression and nuclear fragmentation by fluorescent staining of the DNA. Cells were fixed with 3.7% formaldehyde, stained with DAPI and examined by fluorescence microscopy. Mononucleate cells are either vegetative cells or meiotic cells prior to anaphase I. White arrows indicate binucleate MI cells. Orange arrowheads indicate tetranucleate MII cells and magenta arrows indicate cells with fragmented nuclei that contain >4 DAPI foci. The first three images are wild-type cells (NH716) while the last image is from a *mek1∆ sen1-md* diploid (NH2669).
(TIF)

S3 Fig dRNA-seq analysis of *SEN1* and *sen1-md.* (A) Pairwise Pearson correlation coefficients were calculated from log_2_-transformed DESeq2-normalized counts across two *SEN1* and two *sen1-md* dRNA-seq replicates. (B) Volcano plot showing differential gene expression between *SEN1* and *sen1-md* dRNA-seq samples. The *x*-axis represents the log_2_ fold change (FC) (*sen1-md* vs *SEN1*) and the *y*-axis shows the –log_10_ adjusted *p*-value from DESeq2 analysis. Genes meeting the significance threshold (FDR = 0.01 and |log_2_FC| > 1) are highlighted in pink, whereas non-significant genes are in gray. Blue dots mark genes of particular interest. Pink bars along the axes indicate genes outside the plotted range. A total of 5,496 genes including the noncoding RNAs *IRT1* and *IRT2* were analyzed, with regions defined by their TSS and TTS. (C) Heatmaps of normalized read coverage (count per million) for *SEN1* (left) and *sen1-md* (right) were plotted across 5,491 annotated ORF transcript regions that contain the 5’ and 3’ untranslated regions (ORT-Ts). Each ORF-T was scaled to equal length and aligned at its TSS and TTS, with 500 bp of flanking sequences. Data were sorted according to transcript abundance within the ORF regions. The bottom panel shows line plots of average read coverage for *SEN1* and *sen1-md* across the ORF-T regions shown in the heatmaps. (D) Same as (C), except that the reverse complement (antisense) of each ORF-T was used. The genes were sorted by transcript abundance between -500 bp and TSS. These transcripts include both antisense RNAs that initiated from the 3’ end of ORF-T, as well as readthrough transcripts from adjacent genes.
(TIFF)

S4 Fig Complete Southern blot from which the DSB portion used for Fig 5D. DNA from *SEN1*, *sen1-md* and *P*_*REC8*_*-SEN1* diploids was digested with XhoI and probed to detect recombination at the *HIS4LEU2* hotspot. This digest detects COs and DSBs. The DSB portion is shown in Fig 5D. The CO1 and CO2 bands are located close to the parental bands and can be seen in the lighter exposure of the blot. To detect the DSB bands, the blot was over exposed. The asterisk indicates a band containing a flanking XhoI site gene conversion.
(TIF)

S5 Fig CO/NCO analysis at the *HIS4LEU2* hotspot after 8 and 12 hours in Spo medium. Five biological replicates for *SEN1*, *sen1-md* and *P*_*REC8*_*-SEN1* were transferred to Spo medium and cells were fixed after 8 and 12 hours for physical analysis. (A) Sporulation of the five replicates. (B) Spore viability of the five replicates. (C) Southern blot of DNA was digested with XhoI/NgoMIV and probed to detect recombinants at the *HIS4LEU2* hotspot at described in Fig 5. (D) Quantification of %CO2. (E) Quantification of %NCO1. For A and B, statistical significance was determined using the Mann-Whitney test (** = *p* < 0.008). ns = not significant. For D and E, statistical significance was determined using an unpaired, two-tailed Student’s t test (* = p < -.042).
(TIF)

S6 Fig Flow cytometry analysis of premeiotic DNA synthesis. Meiotic timecourses using *SEN1*, *sen1-md*, *spo11∆* and *sen1-md spo11∆* diploids (*n* = 2) were performed and cells from the indicated timepoints were analyzed for DNA content by flow cytometry. Numbers indicate the hours in Spo medium. Cells between the G1 and G2 peaks are in premeiotic S phase.
(TIF)
