# Supplementary material for: The conserved SEN1 DNA/RNA helicase has multiple functions during yeast meiosis
Source: PLoS Genet. 2025 Dec 11;21(12):e1011684. doi: 10.1371/journal.pgen.1011684 (PMC12714266; doi:10.1371/journal.pgen.1011684)
Supplement: S1 Table — (DOCX) [file pgen.1011684.s010.docx]

**Table S1. *Saccharomyces cerevisiae* strains**

| **Strain** | **Genotype** | **Source** |
| --- | --- | --- |
| NH716 | *MAT****a*** *leu2::hisG HIS4::LEU2-(Bam + ori) ho::hisG ura3(∆Sma-Pst)*  *MATα leu2::hisG HIS4::LEU2-(NgoMIV + ori) ho::hisG ura3(∆Sma-Pst)* | ([Callender and Hollingsworth 2010](#_ENREF_1)) |
| NH729 | Same as NH716 only *mek1∆::natMX4*  *mek1∆::natMX4* | ([Callender and Hollingsworth 2010](#_ENREF_1)) |
| NH1054 | Same as NH716 only *sae2∆::kanMX6*  *sae2∆::kanMX6* | ([Callender and Hollingsworth 2010](#_ENREF_1)) |
| NH1055 | Same as NH716 only *spo11∆::natMX4*  *spo11∆::natMX4* | ([Chen *et al.* 2015](#_ENREF_3)) |
| NH2188 | Same as NH716 only *ndt80∆::natMX4*  *ndt80∆::natMX4* | ([Liu *et al.* 2014](#_ENREF_5)) |
| AM6697 | Same as NH716 only *ndt80∆::hphMX4 kanMX6*::*P_CLB2_-3XHA-SEN1* (*sen1-md)*  *ndt80∆::hphMX4 kanMX6*::*P_CLB2_-3XHA-SEN1* | This work |
| NH2667 | Same as NH716 only *kanMX6*::*P_CLB2_-3XHA-SEN1*  *kanMX6*::*P_CLB2_-3XHA-SEN1* | This work |
| NH2667::pRS306 | Same as NH716 only  *kanMX6*::*P_CLB2_-3XHA-SEN1*  *ura3(∆Sma-Pst)::URA3*  *kanMX6*::*P_CLB2_-3XHA-SEN1 ura3(∆Sma-Pst)* | This work |
| NH2667::pNH410 | Same as NH716 only  *kanMX6*::*P_CLB2_-3XHA-SEN1::URA3:: P_REC8_-SEN1*  *kanMX6*::*P_CLB2_-3XHA-SEN1* | This work |
| NH2667::pBG27 | Same as NH716 only  *kanMX6*::*P_CLB2_-3XHA-SEN1::URA3::P_REC8_-sen1-∆N*  *kanMX6*::*P_CLB2_-3XHA-SEN1* | This work |
| NH2667::pNH317^2^ | Same as NH716 only  *kanMX6*::*P_CLB2_-3XHA-SEN1* *NDT80::URA3::NDT80-mid* | This work |
| NH2667::pNH410::pNH317 | Same as NH716 only  *kanMX6*::*P_CLB2_-3XHA-SEN1::URA3::P_REC8_-SEN1*  *NDT80::URA3::NDT80-mid*  *NDT80* | This work |
| NH2667::pBG28 | Same as NH716 only *kanMX6*::*P_CLB2_-3XHA-SEN1::URA3::P_SEN1_-SEN1*  *kanMX6*::*P_CLB2_-3XHA-SEN1* | This work |
| NH2669 | Same as NH716 only  *kanMX6*::*P_CLB2_-3XHA-SEN1 mek1∆::natMX4* | This work |
| NH2669::pNH410 | Same as NH716 only *kanMX6*::*P_CLB2_-3XHA-SEN1::URA3::P_REC8_-SEN1 mek1∆::natMX4*  *kanMX6*::*P_CLB2_-3XHA-SEN1 mek1∆::natMX4* | This work |
| NH2669::pBG28 | Same as NH716 only *kanMX6*::*P_CLB2_-3XHA-SEN1::URA3:: P_SEN1_-SEN1 mek1∆::natMX4*  *kanMX6*::*P_CLB2_-3XHA-SEN1 mek1∆::natMX4* | This work |
| NH2689 | Same as NH716 only *kanMX6*::*P_CLB2_-3XHA-SEN1 spo11∆::natMX4* | This work |
| NH2689::pNH410 | Same as NH716 only  *kanMX6*::*P_CLB2_-3XHA-SEN1::URA3::P_REC8_-SEN1 spo11∆::natMX4*  *kanMX6*::*P_CLB2_-3XHA-SEN1 spo11∆::natMX4* | This work |
| NH2689::pBG28 | Same as NH716 only  *kanMX6*::*P_CLB2_-3XHA-SEN1::URA3::P_SEN1_-SEN1 spo11∆::natMX4*  *kanMX6*::*P_CLB2_-3XHA-SEN1 spo11∆::natMX4* | This work |
| NH2473 | Same as NH716 only *trp1-5’∆::hphMX4*  *trp1-5’∆::hphMX4* | This work |
| NH2520 | Same as NH716 only *kanMX6*::*P_CLB2_-3XHA-SEN1*  *trp1-5’∆::hphMX4* | This work |
| NH2846 | Same as NH716 only *kanMX6*::*P_CLB2_-3XHA-SEN1*  *sae2∆::natMX4* | This work |
| NH2846::pNH410 | Same as NH716 only *kanMX6*::*P_CLB2_-3XHA-SEN1::URA3::P_REC8_-SEN1 sae2∆::natMX4* | This work |
| NH2081 | *MAT***a** *leu2ΔhisG his4-x ARG4 ndt80Δ::hphMX4 lys2 hoΔLYS2 ura3*  *MATα leu2-k HIS4 arg4-Nsp ndt80Δ::hphMX4 lys2 hoΔLYS2 ura3* | ([Chen *et al.* 2018](#_ENREF_2)) |
| NH2081::pNH317^2^ | Same as NH2081 only *ura3::NDT80-mid::URA3*  *ura3::NDT80-mid::URA3* | This work |
| NH2843 | *MAT****a*** *leu2 HIS4::LEU2-(Bam + ori) arg4-Nsp ho ura3 sml1∆::3XHA*  *MATα leu2 HIS4 arg4-Nsp ho ura3 sml1∆::3XHA*  *rad53∆::kanMX6 kanMX6::P_CLB2_-SEN1*  *rad53∆::kanMX6 kanMX6::P_CLB2_-SEN1* | This work |
| NH2844 | *MAT****a*** *leu2 HIS4::LEU2-(Bam + ori) arg4-Nsp ho ura3 sml1∆::3XHA*  *MATα leu2 HIS4 arg4-Nsp ho ura3 sml1∆::3XHA*  *rad53∆::kanMX6 kanMX6::P_CLB2_-SEN1 spo11∆::natMX4*  *rad53∆::kanMX6 kanMX6::P_CLB2_-SEN1 spo11∆::natMX4* | This work |
| S2683 | *MATα leu2u-k arg4-Nsp ura3 lys2 ho::LYS2* | ([de los Santos and Hollingsworth 1999](#_ENREF_4)) |
| 39823 | *MAT***a** *leu2::hisG ho::LYS2 lys2 trp1::hisG P_CUP1_-IME1::natMX* | Gloria Brar |
| 39806 | *MATα* *leu2::hisG ho::LYS2 lys2 trp1::hisG P_CUP1_-IME1::natMX* | Gloria Brar |
| 39807 | *MAT****a*** *leu2::hisG ho::LYS2 lys2 trp1::hisG P_CUP1_-IME1::natMX*  *MATα* *leu2::hisG ho::LYS2 lys2 trp1::hisG P_CUP1_-IME1::natMX* | Gloria Brar |
| NH2842 | Same as 39807 only *kanMX6::P_CLB2_-SEN1*  *kanMX6::P_CLB2_-SEN1* | This work |
| NH2842::pBG45 | Same as 39807 only *kanMX6::P_CLB2_-SEN1::TRP1-P_REC8_-SEN1*  *kanMX6::P_CLB2_-SEN1* | This work |
| LZY2919 | *MAT***a** *ZIP3-13myc::hph URA3::P_CYC1_-lacI-GFP scp1(Ch XV telomere)::lacO-LEU2*  *MATα ZIP3-13myc::hph URA3::P_CYC1_-lacI-GFP scp1(Ch XV telomere)::lacO-LEU2*  *rnh1∆::kanMX6 rnh201 rnh201∆::natNT2 hpr1∆::kanMX6*  *rnh1∆::kanMX6 rnh201 rnh201∆::natNT2 hpr1∆::kanMX6* | ([Yang *et al.* 2021](#_ENREF_6)) |

Callender, T. L., and N. M. Hollingsworth, 2010 Mek1 suppression of meiotic double-strand break repair is specific to sister chromatids, chromosome autonomous and independent of Rec8 cohesin complexes. Genetics 185**:** 771-782.

Chen, X., R. Gaglione, T. Leong, L. Bednor, T. de Los Santos *et al.*, 2018 Mek1 coordinates meiotic progression with DNA break repair by directly phosphorylating and inhibiting the yeast pachytene exit regulator Ndt80. PLoS Genet 14**:** e1007832.

Chen, X., R. T. Suhandynata, R. Sandhu, B. Rockmill, N. Mohibullah *et al.*, 2015 Phosphorylation of the synaptonemal complex protein Zip1 regulates the crossover/noncrossover decision during yeast meiosis. PLoS Biol 13**:** e1002329.

de los Santos, T., and N. M. Hollingsworth, 1999 Red1p: A *MEK1*-dependent phosphoprotein that physically interacts with Hop1p during meiosis in yeast. J. Biol. Chem. 274**:** 1783-1790.

Liu, Y., W. A. Gaines, T. Callender, V. Busygina, A. Oke *et al.*, 2014 Down-regulation of Rad51 activity during meiosis in yeast prevents competition with Dmc1 for repair of double-strand breaks. PLoS Genet 10**:** e1004005.

Yang, X., B. Zhai, S. Wang, X. Kong, Y. Tan *et al.*, 2021 RNA-DNA hybrids regulate meiotic recombination. Cell Rep 37**:** 110097.
