# Supplementary material for: The conserved SEN1 DNA/RNA helicase has multiple functions during yeast meiosis
Source: PLoS Genet. 2025 Dec 11;21(12):e1011684. doi: 10.1371/journal.pgen.1011684 (PMC12714266; doi:10.1371/journal.pgen.1011684)
Supplement: S2 Table — (DOCX) [file pgen.1011684.s011.docx]

**S2 Table. Plasmids**

| **Plasmid name** | **Relevant yeast genotype** | **Source** |
| --- | --- | --- |
| pRS306 | *URA3* | ([Sikorski and Hieter 1989](#_ENREF_7)) |
| pRS304 | *TRP1* | ([Sikorski and Hieter 1989](#_ENREF_7)) |
| p4339 | *natMX4* | ([Goldstein and McCusker 1999](#_ENREF_2)) |
| pAG32 | *hphMX4* | ([Goldstein and McCusker 1999](#_ENREF_2)) |
| pFA6a-kanMX6 | *kanMX6* | ([Longtine *et al.* 1998](#_ENREF_5)) |
| pMJ787 | *kanMX6::P_CLB2_-3xHA* | ([Lee and Amon 2003](#_ENREF_4)) |
| pMPY-3HA | *3HA-URA3-3HA* | ([Schneider *et al.* 1995](#_ENREF_6)) |
| 2µ *SEN1* | *2µ LEU2 SEN1* | ([Jones *et al.* 2008](#_ENREF_3)) |
| pNH257 | *P_REC8_ URA3* | ([Ziesel *et al.* 2022](#_ENREF_8)) |
| pNH410 | *P_REC8_-SEN1 URA3* | This work |
| pBG45 | *P_REC8_-SEN1 TRP1* | This work |
| pBG27 | *P_REC8_-sen1^1004-2231^ (∆N) URA3* | This work |
| pNH317 | *NDT80-mid URA3* | ([Chen *et al.* 2018](#_ENREF_1)) |
| pBG28 | *P_SEN1_-SEN1 URA3* | This work |

Chen, X., R. Gaglione, T. Leong, L. Bednor, T. de Los Santos *et al.*, 2018 Mek1 coordinates meiotic progression with DNA break repair by directly phosphorylating and inhibiting the yeast pachytene exit regulator Ndt80. PLoS Genet 14**:** e1007832.

Goldstein, A. L., and J. H. McCusker, 1999 Three new dominant drug resistance cassettes for gene disruption in *Saccharomyces cerevisiae*. Yeast 15**:** 1541-1553.

Jones, G. M., J. Stalker, S. Humphray, A. West, T. Cox *et al.*, 2008 A systematic library for comprehensive overexpression screens in Saccharomyces cerevisiae. Nature methods 5**:** 239-241.

Lee, B. H., and A. Amon, 2003 Role of Polo-like kinase CDC5 in programming meiosis I chromosome segregation. Science 300**:** 482-486.

Longtine, M. S., A. McKenzie, 3rd, D. J. Demarini, N. G. Shah, A. Wach *et al.*, 1998 Additional modules for versatile and economical PCR-based gene deletion and modification in *Saccharomyces cerevisiae*. Yeast 14**:** 953-961.

Schneider, B. L., W. Seufert, B. Steiner, Q. H. Yang and A. B. Futcher, 1995 Use of polymerase chain reaction epitope tagging for protein tagging in Saccharomyces cerevisiae. Yeast 11**:** 1265-1274.

Sikorski, R. S., and P. Hieter, 1989 A system of shuttle vectors and yeast host strains designed for efficient manipulation of DNA in *Saccharomyces cerevisiae*. Genetics 122**:** 19-27.

Ziesel, A., Q. Weng, J. S. Ahuja, A. Bhattacharya, R. Dutta *et al.*, 2022 Rad51-mediated interhomolog recombination during budding yeast meiosis is promoted by the meiotic recombination checkpoint and the conserved Pif1 helicase. PLoS Genet 18**:** e1010407.
