# Supplementary material for: The conserved SEN1 DNA/RNA helicase has multiple functions during yeast meiosis
Source: PLoS Genet. 2025 Dec 11;21(12):e1011684. doi: 10.1371/journal.pgen.1011684 (PMC12714266; doi:10.1371/journal.pgen.1011684)
Supplement: S3 Table — (DOCX) [file pgen.1011684.s012.docx]

**S3 Table. Antibodies**

| Antigen | Primary | Purpose | Dilution | Source | Secondary | Dilution | Source |
| --- | --- | --- | --- | --- | --- | --- | --- |
| Arp7 | α -Arp7 | Immunoblot | 1:50,000 | Santa Cruz sc-8961 | α-Goat | 1:15,000 | Santa Cruz sc-2354 |
| pHed1 | α -pT40 Hed1 | Immunoblot | 1:20,000 | N. M Hollingsworth | α -Rabbit | 1:10,000 | Invitrogen 31460 |
| Hop1 | α -Hop1 | Immunoblot | 1:10,000 | N. M Hollingsworth | α -Rabbit | 1:10,000 | Invitrogen 31460 |
| Mek1 | α -Mek1 | Immunoblot | 1:10,000 | N. M. Hollingsworth | α -Guinea Pig | 1:10,000 | Santa Cruz sc-2903 |
| Rec8 | α -Rec8 | Immunoblot | 1:50,000 | N. M. Hollingsworth | α -Guinea Pig | 1:10,000 | Santa Cruz sc-2903 |
| Red1 | α -GST-Red1 | Immunoblot | 1:10,000 | N. M Hollingsworth | α -Rabbit | 1:10,000 | Invitrogen 31460 |
| Red1 | α -GST-Red1 | Immunostaining – Figure 1 | 1:200 | N. M Hollingsworth | α -Rabbit – Alexa 488 | 1:1,000 | Invitrogen A11008 |
| Red1 | α -Red1 | Immunostaining – Figure 5 | 1:200 | G. S. Roeder | α -Rabbit – Alexa 488 | 1:200 | Jackson – 711-545-152 |
| Gmc2 | α -Gmc2 | Immunostaining  – Figure 5 | 1:800 | A. J. MacQueen | α -mouse – Alexa 594 or  α -mouse – Alexa 488 | 1:200 | Jackson- 715-585-150 or 715-545-150 |
| Zip1 | α -Zip1 | Immunostaining | 1:150 | A. J. MacQueen | α -Rabbit – Alexa 594 | 1:200 | Jackson – 711-585-152 |
| S9.6 | α -RNA – DNA hybrid | Immunostaining | 1:1000 | Kerafast - ENH001 | α -mouse – Alexa 594 | 1:10,000 | Invitrogen A32742 |
| Sen1 | α -Sen1 | Immunoblot | 1:5,000 | N. M Hollingsworth | α -Guinea Pig | 1:10,000 | Santa Cruz sc-2903 |
