# Supplementary material for: The conserved SEN1 DNA/RNA helicase has multiple functions during yeast meiosis
Source: PLoS Genet. 2025 Dec 11;21(12):e1011684. doi: 10.1371/journal.pgen.1011684 (PMC12714266; doi:10.1371/journal.pgen.1011684)
Supplement: S4 Table — (DOCX) [file pgen.1011684.s013.docx]

**S4 Table. Primers for amplifying ribosomal DNA sequences**

| Name | Alias | Sequence (5’-3’)^a^ |
| --- | --- | --- |
| **25S rDNA 5’ end** | | |
| EL1700 | RDN25-1F | GTTTGACCTCAAATCAGGTAGGAGTACC |
| EL1726 | RDN25-5’end-R-T7 | aattctaatacgactcactatagggagaGGAACGGCCCCAAAGTTGC |
| **25S rDNA 3’ end** | | |
| EL1701 | RDN25-1R-T7 | aattctaatacgactcactatagggagaACAAATCAGACAACAAAGGCTTAATCTCAG |
| EL1727 | RDN25-3’end-F | GAACCATAGCAGGCTAGCAACG |
| **18S rDNA 5’ end** | | |
| EL1702 | RDN18-1F | TATCTGGTTGATCCTGCCAGTAGTC |
| EL1728 | RDN18-5’endR-T7 | aattctaatacgactcactatagggagaCCAAAGGGTCGAGATTTTAAGCATGT |
| **18S rDNA 3’ end** | | |
| EL1703 | RDN18-1R-T7 | aattctaatacgactcactatagggagaTAATGATCCTTCCGCAGGTTCACCTA |
| EL1729 | RDN18-3’end-F | GCGTTGATTACGTCCCTGCC |
| **5.8 rDNA** | | |
| EL1704 | RDN58-1F | AAACTTTCAACAACGGATCTCTTGGTT |
| EL1705 | RDN58-1R-T7 | aattctaatacgactcactatagggagaAAATGACGCTCAAACAGGCATGC |
| **5.8 rDNA** | | |
| EL1706 | RDN5-1F | GGTTGCGGCCATATCTACCAGAAA |
| EL1707 | RDN5-1R-T7 | aattctaatacgactcactatagggagaAGATTGCAGCACCTGAGTTTC |

^a^lower case letters indicate the T7 promoter sequence, upper case letters indicate regions homologous to the indicated rDNA gene
