# Supplementary figures and images for: The conserved SEN1 DNA/RNA helicase has multiple functions during yeast meiosis
Source: PLoS Genet. 2025 Dec 11;21(12):e1011684. doi: 10.1371/journal.pgen.1011684 (PMC12714266; doi:10.1371/journal.pgen.1011684)

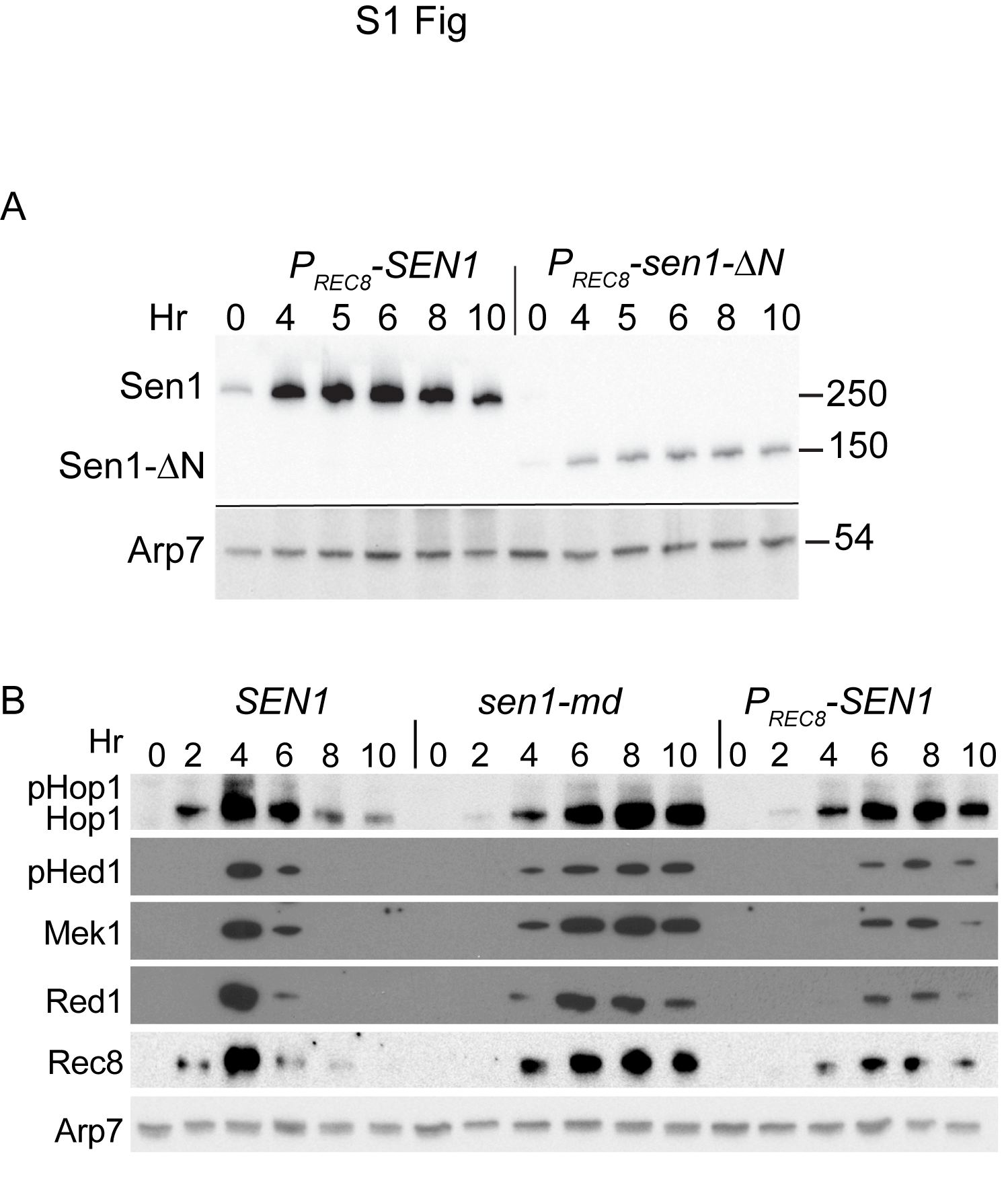

Supplement: S1 Fig — (A) The sen1-md diploid, NH2667, was transformed with an integrating plasmid containing either PREC8-SEN1 (pNH410) or PREC8-sen1-∆N (pBG27) and induced to undergo meiosis. Protein samples from the indicated timepoints were probed with α-Sen1 antibodies. α-Arp7 antibodies were used to detect Arp7 as a loading control. Numbers on the right indicate the positions of molecular weight markers in kiloDaltons. The black line indicates that the same samples were run on two different gels and probed with different antibodies. “Hr” refers to hours in Spo medium. (B) Timing of expression of various meiosis-specific proteins. Protein extracts from meiotic timecourses using SEN1, sen1-md and PREC8-SEN1 strains were probed with antibodies against the indicated proteins. “pHop” and “pHed1” indicate phosphorylated Hop1 and Hed1, respectively. (TIF) [file pgen.1011684.s014.tif]

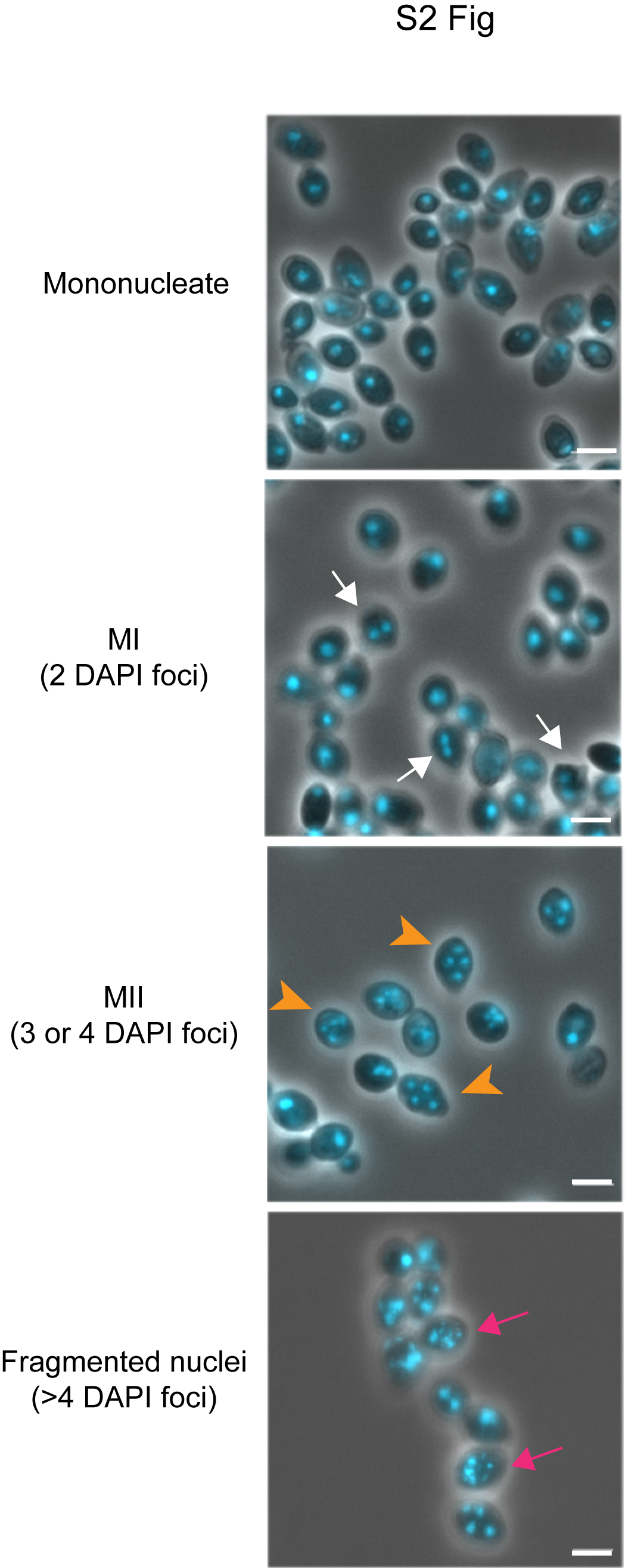

Supplement: S2 Fig — Cells were fixed with 3.7% formaldehyde, stained with DAPI and examined by fluorescence microscopy. Mononucleate cells are either vegetative cells or meiotic cells prior to anaphase I. White arrows indicate binucleate MI cells. Orange arrowheads indicate tetranucleate MII cells and magenta arrows indicate cells with fragmented nuclei that contain >4 DAPI foci. The first three images are wild-type cells (NH716) while the last image is from a mek1∆ sen1-md diploid (NH2669). (TIF) [file pgen.1011684.s015.tif]

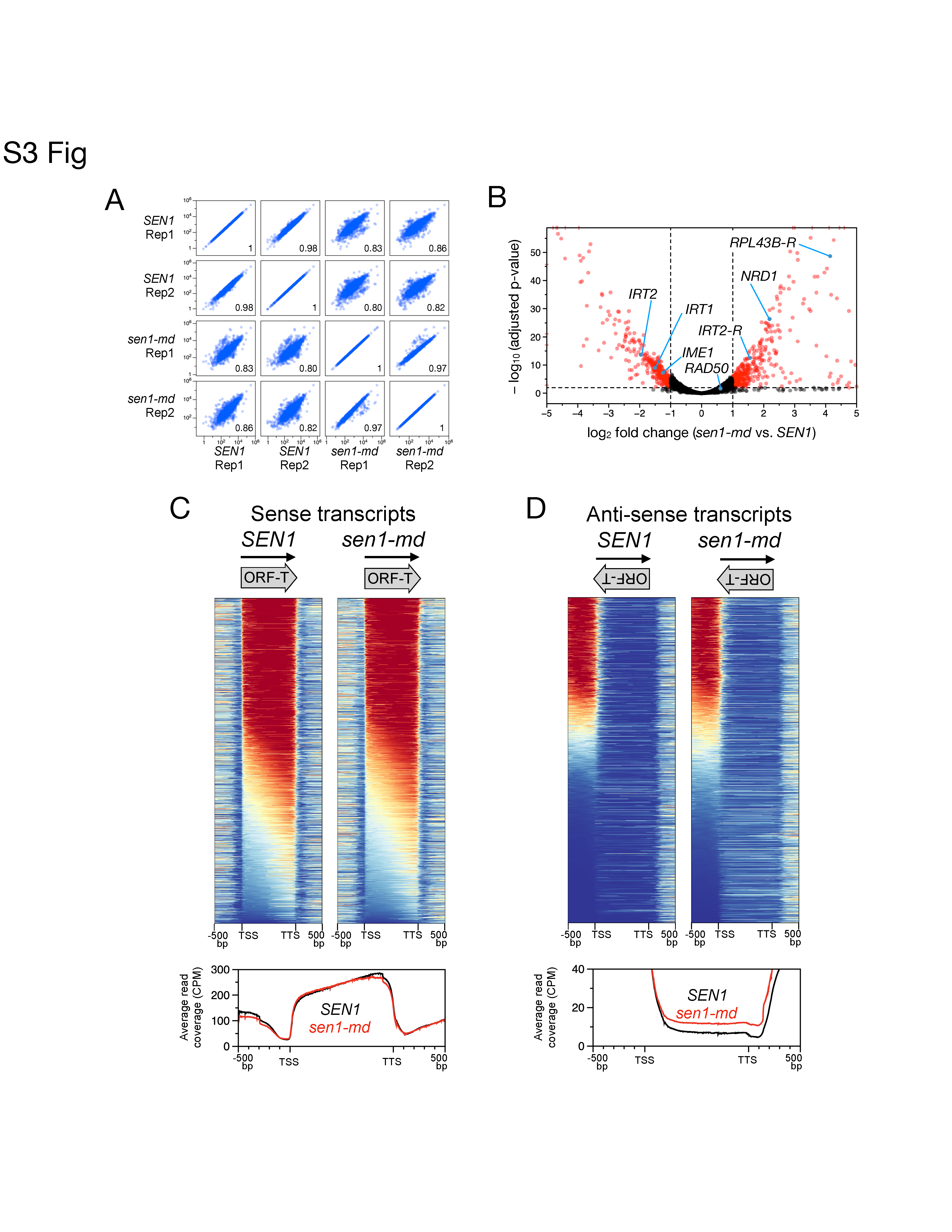

Supplement: S3 Fig — (A) Pairwise Pearson correlation coefficients were calculated from log2-transformed DESeq2-normalized counts across two SEN1 and two sen1-md dRNA-seq replicates. (B) Volcano plot showing differential gene expression between SEN1 and sen1-md dRNA-seq samples. The x-axis represents the log2 fold change (FC) (sen1-md vs SEN1) and the y-axis shows the –log10 adjusted p-value from DESeq2 analysis. Genes meeting the significance threshold (FDR = 0.01 and |log2FC| > 1) are highlighted in pink, whereas non-significant genes are in gray. Blue dots mark genes of particular interest. Pink bars along the axes indicate genes outside the plotted range. A total of 5,496 genes including the noncoding RNAs IRT1 and IRT2 were analyzed, with regions defined by their TSS and TTS. (C) Heatmaps of normalized read coverage (count per million) for SEN1 (left) and sen1-md (right) were plotted across 5,491 annotated ORF transcript regions that contain the 5’ and 3’ untranslated regions (ORT-Ts). Each ORF-T was scaled to equal length and aligned at its TSS and TTS, with 500 bp of flanking sequences. Data were sorted according to transcript abundance within the ORF regions. The bottom panel shows line plots of average read coverage for SEN1 and sen1-md across the ORF-T regions shown in the heatmaps. (D) Same as (C), except that the reverse complement (antisense) of each ORF-T was used. The genes were sorted by transcript abundance between -500 bp and TSS. These transcripts include both antisense RNAs that initiated from the 3’ end of ORF-T, as well as readthrough transcripts from adjacent genes. (TIFF) [file pgen.1011684.s016.tiff]

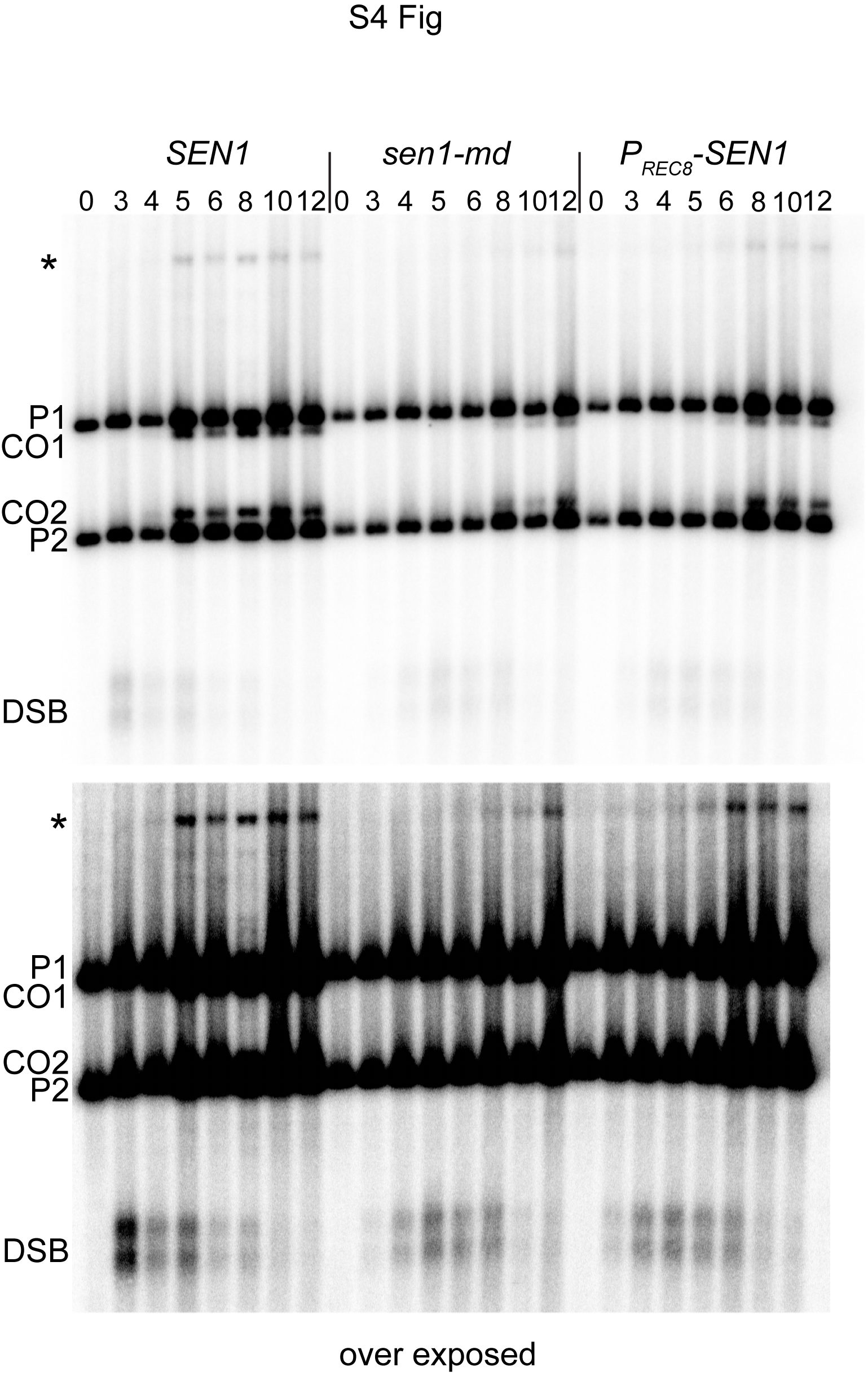

Supplement: S4 Fig — DNA from SEN1, sen1-md and PREC8-SEN1 diploids was digested with XhoI and probed to detect recombination at the HIS4LEU2 hotspot. This digest detects COs and DSBs. The DSB portion is shown in Fig 5D. The CO1 and CO2 bands are located close to the parental bands and can be seen in the lighter exposure of the blot. To detect the DSB bands, the blot was over exposed. The asterisk indicates a band containing a flanking XhoI site gene conversion. (TIF) [file pgen.1011684.s017.tif]

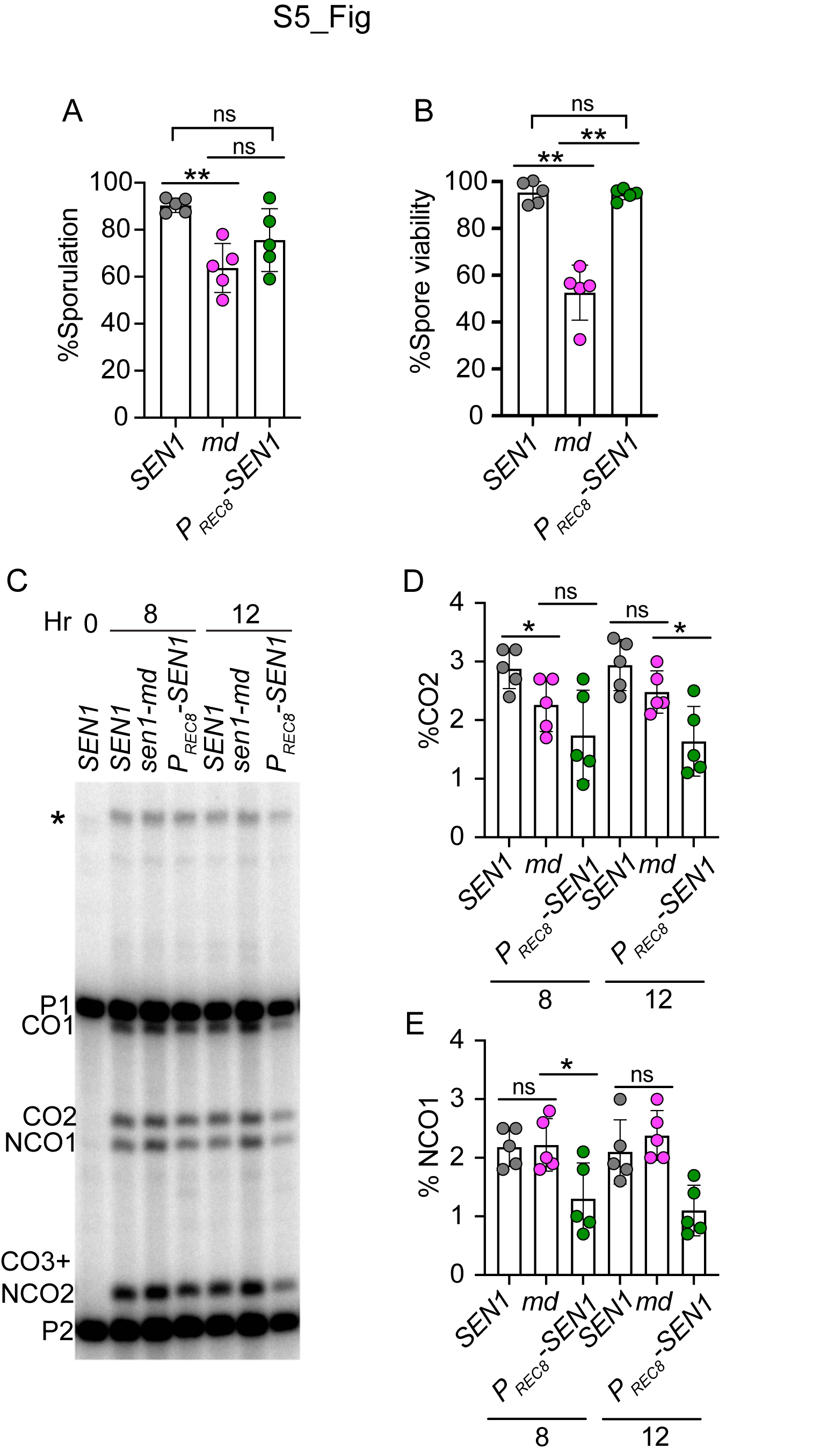

Supplement: S5 Fig — Five biological replicates for SEN1, sen1-md and PREC8-SEN1 were transferred to Spo medium and cells were fixed after 8 and 12 hours for physical analysis. (A) Sporulation of the five replicates. (B) Spore viability of the five replicates. (C) Southern blot of DNA was digested with XhoI/NgoMIV and probed to detect recombinants at the HIS4LEU2 hotspot at described in Fig 5. (D) Quantification of %CO2. (E) Quantification of %NCO1. For A and B, statistical significance was determined using the Mann-Whitney test (** = p < 0.008). ns = not significant. For D and E, statistical significance was determined using an unpaired, two-tailed Student’s t test (* = p < -.042). (TIF) [file pgen.1011684.s018.tif]

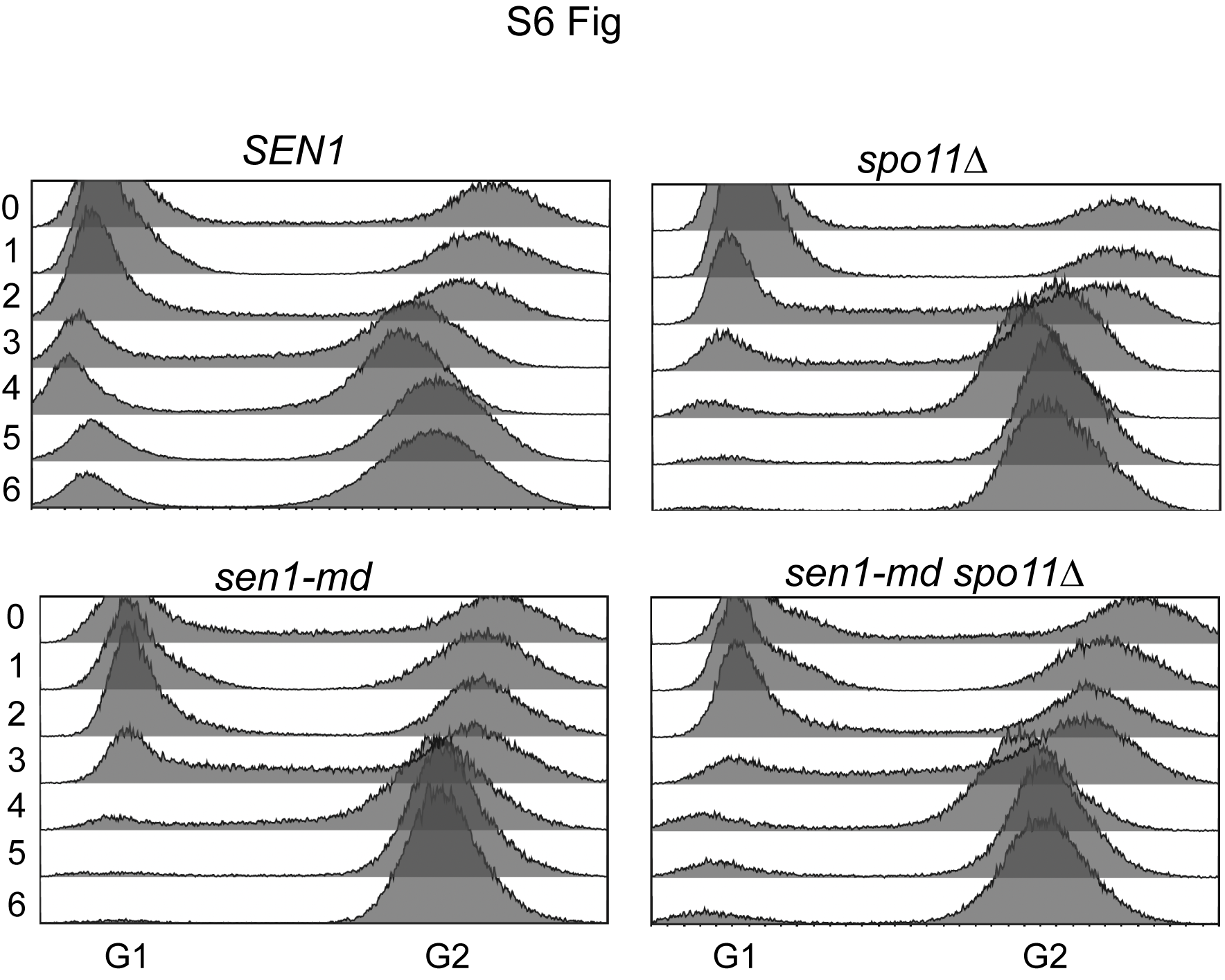

Supplement: S6 Fig — Meiotic timecourses using SEN1, sen1-md, spo11∆ and sen1-md spo11∆ diploids (n = 2) were performed and cells from the indicated timepoints were analyzed for DNA content by flow cytometry. Numbers indicate the hours in Spo medium. Cells between the G1 and G2 peaks are in premeiotic S phase. (TIF) [file pgen.1011684.s019.tif]
